# Supplementary material for: CurT/CURT1 proteins are involved in cell and chloroplast division coordination of cyanobacteria and green algae
Source: Nat Commun. 2025 Sep 25;16:8424. doi: 10.1038/s41467-025-64163-x (PMC12462475; doi:10.1038/s41467-025-64163-x)
Supplement: Supplementary file 1 — Supplementary Information [file 41467_2025_64163_MOESM1_ESM.pdf]

Supplementary Information

CurT/CURT1 Proteins Are Involved in Cell and Chloroplast Division Coordination of Cyanobacteria and Green Algae

Marcel Dann*,♦,1,2,3, Eunchul Kim*,♦,2,4, Konomi Fujimura-Kamada[2], Vjosa Berisha[1], Mami Nomura[5], Anne-Christin Pohland[1], Mai Watanabe[1], Matthias Ostermeier[3], Frederik Sommer[6], Michael Schroda[6], Shin-ya Miyagishima[7], and Jun Minagawa[2]

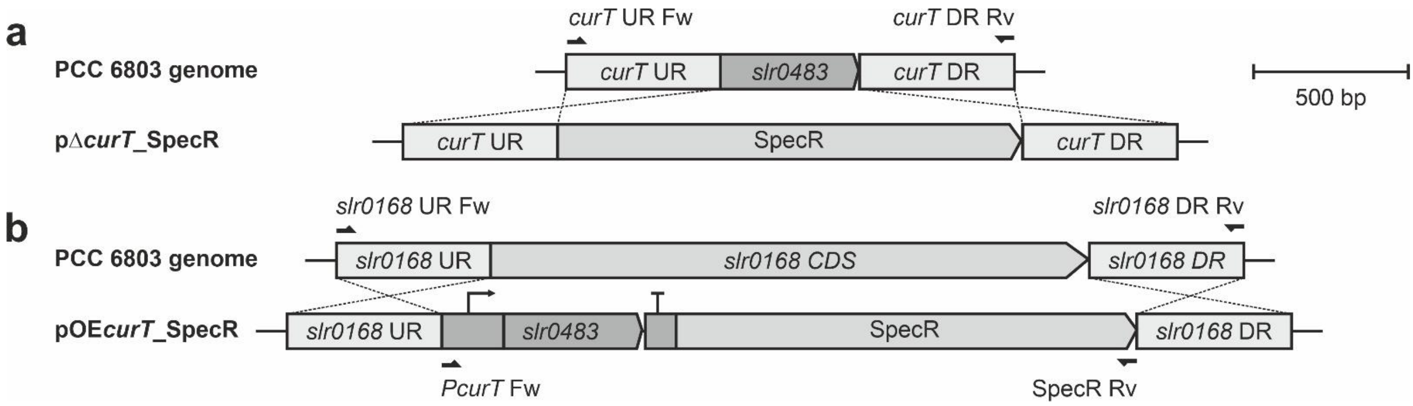

Supplementary Fig. 1: Generation of *Synechocystis* sp. PCC 6803 *curT* expression mutants through homologous recombination. a, Schematic maps of the 188 pΔ*curT* knock-out and the pOE*curT* overexpression constructs used in this study. SpecR, spectinomycin resistance gene *aadA*; UR, upstream (5') region; DR, downstream (3') region; CDS, coding sequence. UR/DR regions used for homologous recombination with genomic *curT* (*slr0483*) and neutral site (*slr0168*) target loci are indicated as grey boxes. Primer-binding sites for genotyping PCR are indicated as half arrows. Constructs are drawn to scale according to scale bar in units of base pairs (bp). **b,** The *curT* coding sequence (ORF *slr0483*) has been replaced with a SpecR resistance cassette, and the entire *curT* gene (200 bp UR region + coding sequence + 100 bp 3' region) has been inserted into the genomic neutral site by homologous recombination. Genotyping PCR confirming partial and complete replacement of all chromosomal curT gene copies in KD and KO cells, as well as the insertion of an additional *curT* gene copy in the *slr0168* locus for *curT* OE strains. WT, wild type.

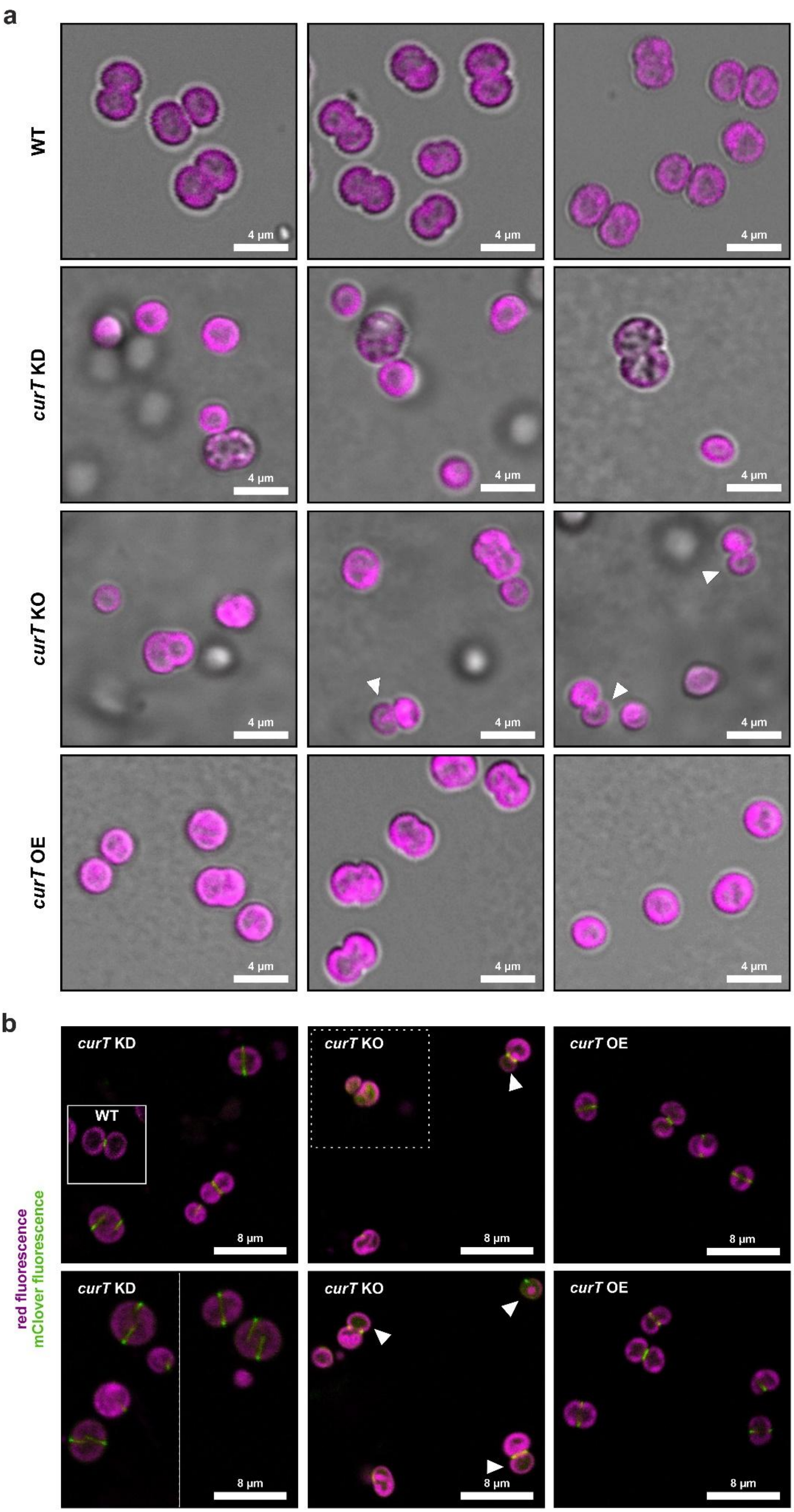
a
WT
curT KD
curT KO
curT OE
4 µm
b
curT KD
WT
curT KO
curT OE
8 µm
red fluorescence
mClover fluorescence

Supplementary Fig. 2: *Synechocystis* sp. PCC 6803 *curT* mutants display asymmetric and impaired cell division. a, Confocal laser scanning micrographs of transmitted light overlayed with chlorophyll *a* and phycobiliprotein red fluorescence of wildtype (WT), *curT* knock-down (KD), knock-out (KO) and overexpression (OE) strain cells grown on solid media. White arrowheads indicate asymmetric thylakoid inheritance. **b,** Representative confocal laser scanning micrographs of green (m-Clover, green) and red (chlorophyll/phycobilin, magenta) fluorescence in *Synechocystis curT* mutant strains expressing an FtsZ-mClover fusion protein. A wildtype (WT) reference is provided (inset). White arrow heads signify *curT* KO daughter cells that inherited reduced thylakoid systems. Confocal microscopic imaging was performed at least twice for two independent clones of each genotype, yielding similar results.

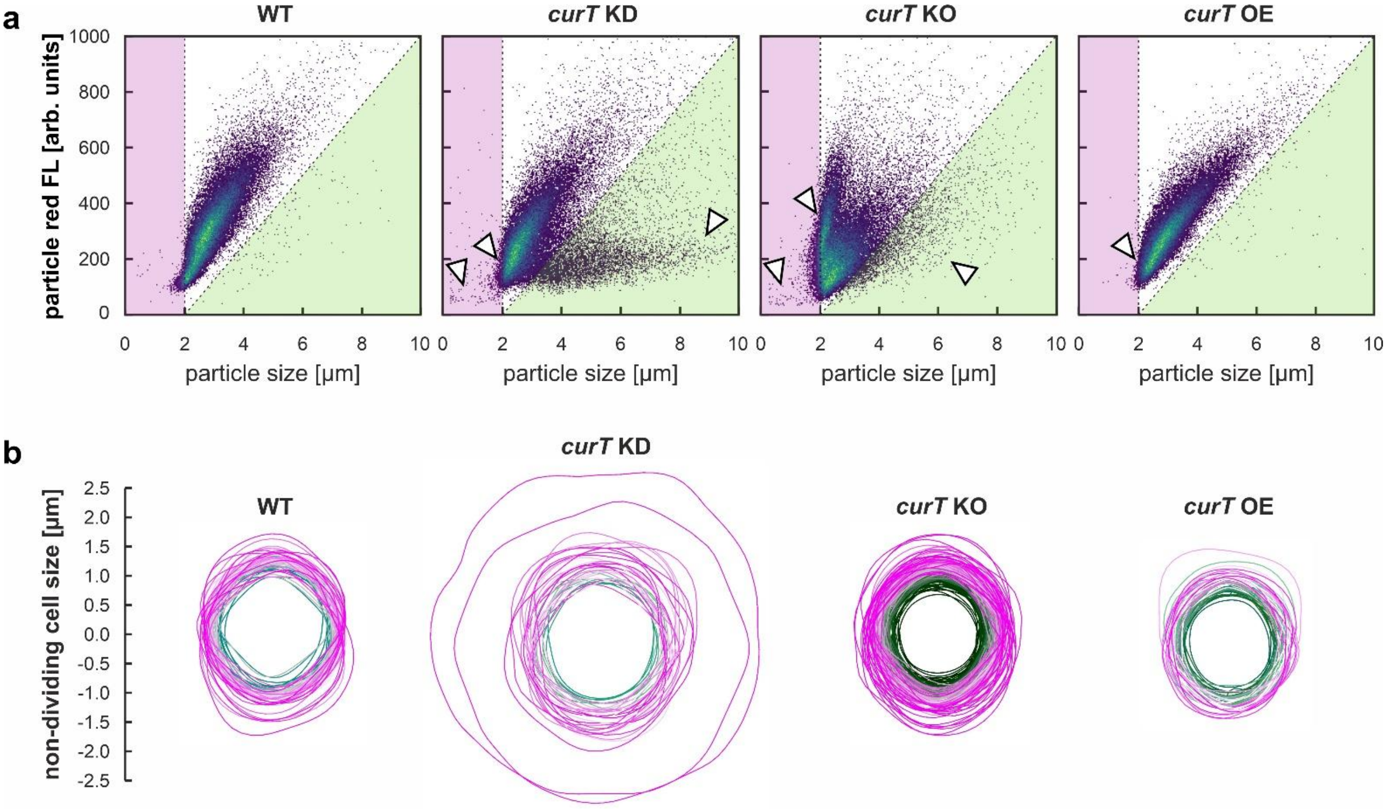

Supplementary Fig. 3: *Synechocystis* cell size is differentially affected by knock-down (KD), knock-out (KO), and overexpression (OE) of *curT*. a, Cell-size distributions of *curT* mutants as estimated by particle sizer. Apparent particle size (x axis) was estimated from forward scatter (FSC) using a size calibration kit (F13838, Invitrogen). Red fluorescence (FL) intensity (primary y axis, arbitrary (arb.) units; excitation 532 nm, emission 680 ± 15 nm) is shown as detector output values without absolute calibration. Dot plots are colored by point density (secondary y axis, arb. units relative to maximum density). Particle size *vs* red fluorescence plots represent data from *n* = 3 biological replicates per genotype. White arrowheads indicate sub-populations absent from the WT control (magenta, mini cell range; green, large low-fluorescent cell range). **b,** Representative cell shape maps of WT, KD, KO, and OE non-dividing cells produced by Fiji MicrobeJ. The experiment was performed independently twice with similar results.

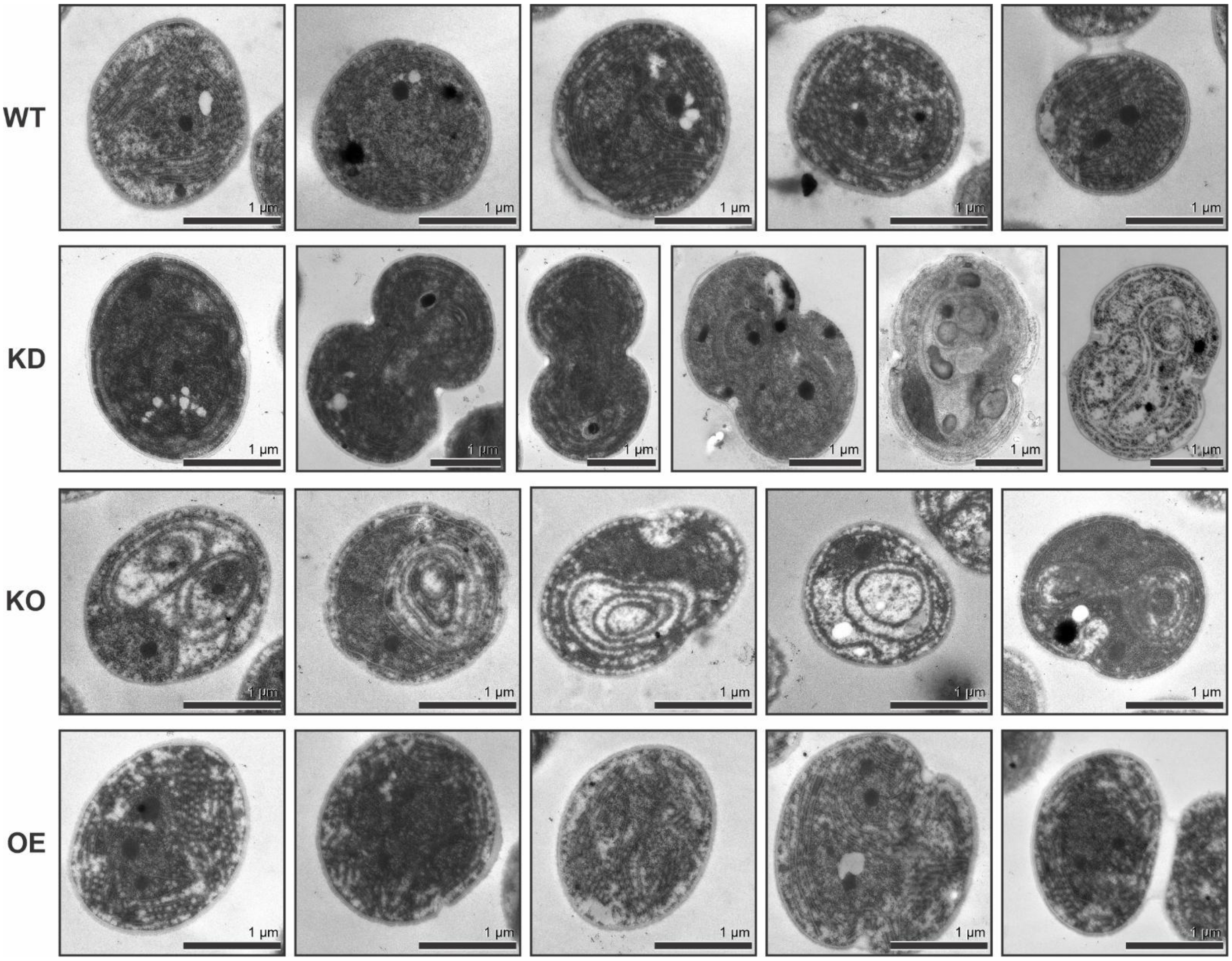

Supplementary Fig. 4: *Synechocystis curT* mutant cell cross-section transmission electron micrographs. Representative transmission electron micrographs of wild type (WT), knock-down (KD), knock-out (KO), and overexpression (OE) strains. For the segregating KD mutant population, the observed phenotypic gradient spanning WT-like cells (left) to severely affected cells of large size and pronounced thylakoid system distortion (right) is presented. Transmission electron micrographs are representative of two biological replicates and *n* = 11/11/13/12 individual cells of WT, KD, KO, and OE, respectively.

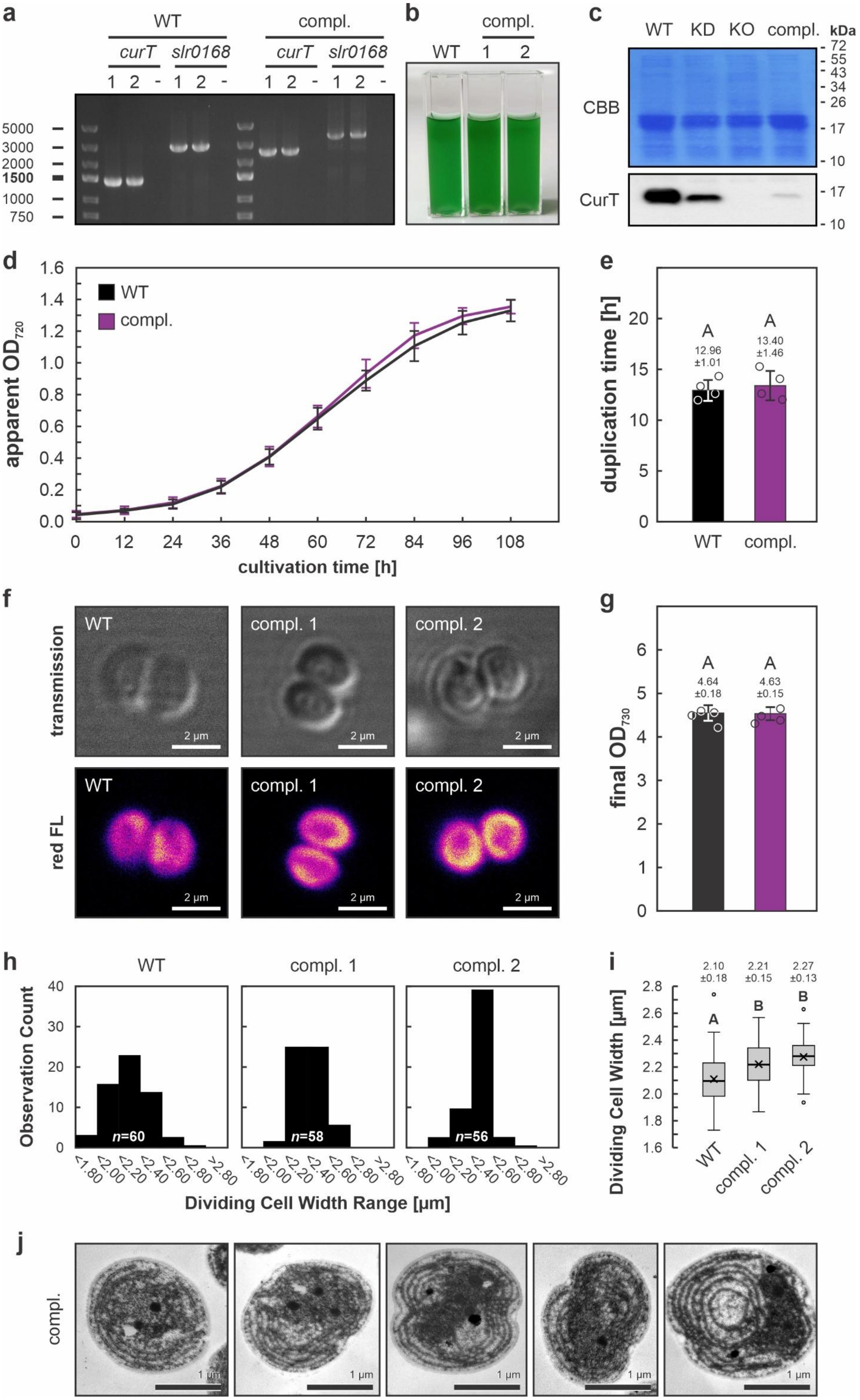
a
WT
compl.
curT
slr0168
1 2 -
5000
3000
2000
1500
1000
750
b
WT
compl.
1 2
c
WT KD KO compl.
kDa
72
55
43
34
26
17
10
CBB
CurT
d
apparent OD720
cultivation time [h]
WT
compl.
e
duplication time [h]
A
12.96
±1.01
A
13.40
±1.46
WT
compl.
f
transmission
red FL
WT
compl. 1
compl. 2
2 µm
g
final OD730
A
4.64
±0.18
A
4.63
±0.15
h
Observation Count
WT
compl. 1
compl. 2
n=60
n=58
n=56
Dividing Cell Width Range [µm]
i
Dividing Cell Width [µm]
2.10
±0.18
2.21
±0.15
2.27
±0.13
A
B
B
WT
compl. 1
compl. 2
j
compl.
1 µm

Supplementary Fig. 5: The cell division defect of *Synechocystis* Δ*curT* mutants can be complemented through introduction of a *curT* gene copy into the neutral site locus *slr0168*. **a,** To obtain *curT* complementation (compl.) strains, the *curT* coding sequence (ORF *slr0483*) was replaced with a spectinomycin resistance cassette, and the entire *curT* gene (200 bp UR region + coding sequence + 100 bp 3' region) was inserted into the *slr0168* genomic neutral site by homologous recombination. Genotyping PCR confirms complete replacement of endogenous locus *curT* and *slr0168* gene copies with the corresponding mutant alleles. For expected amplicon sizes, see Fig. 1a. **b,** Representative cell culture phenotypes of WT and compl. strains recorded at 108 hours past inoculation (hpi). **c**, Immunoblot analysis confirming accumulation of cellular CurT in compl. mutant strain. KD, knock-down. KO, knock-out. CBB, Coomassie brilliant blue stain of PVDF membrane provided as loading control. **d**, Growth curves of WT and compl. strains cultivated for 108 h at 25 °C and 50 µmol photons m^{-2} s^{-1} recorded as optical density (OD) at wavelength λ = 720 nm (apparent OD_{720}; PSI Multicultivator inbuilt photometer). Curves represent average values for *n* = 4 biological replicates ± standard deviation (SD; error bars) **e**, Exponential growth phase cell duplication times derived from growth curves shown in (**d**). Data points represent means, error bars represent standard deviations of *n* = 4 biological replicates. **f**, Representative confocal laser scanning micrographs of WT and two independent compl. mutants. Magenta, chlorophyll *a* and phycobilin red fluorescence (red FL). **g**, Final OD at λ = 730 nm of cultures shown in (**b**, **d**). Chart represents average values for *n* = 4 biological replicates ± standard deviation (SD; error bars). Uppercase letters indicate statistically significant differences ($p \leq 0.05$) according to Student's t-test (two-sided; p = 0.91). **h**, Dividing-cell width distributions (measured parallel to division plane). **i**, Dividing-cell width distributions. Boxplot centre line = median; cross = mean; boxes = 25th–75th percentiles; whiskers = 1.5 × IQR; circles = outliers; all datapoints shown. Values below plots indicate mean ± SD. Precise sample sizes (number of measured cells *n*) are given in panel (**h**). Numbers below boxplot correspond to average values in [µm] ± standard deviation. Uppercase letters indicate statistically significant differences ($p \leq 0.05$) according to multiple simultaneous comparisons in *post hoc* Bonferroni-Holm-corrected Tukey HSD tests after significant among-group differences were detected by one-way ANOVA (two-sided; $p = 2.20\times10^{-7}$). **j**, Representative transmission electron micrographs of *curT* compl. cell cross-sections. ***Note:*** Data shown in panels **f**, **g**, and **h** represents chemically fixated cell-material, resulting in a slight overall reduction in observed cell sizes as compared to data shown in **Fig. 1**. Experiments were performed with two independently transformed *curT* complementation strains. Transmission electron micrographs are representative of two biological replicates and *n* = 9 individual cells.

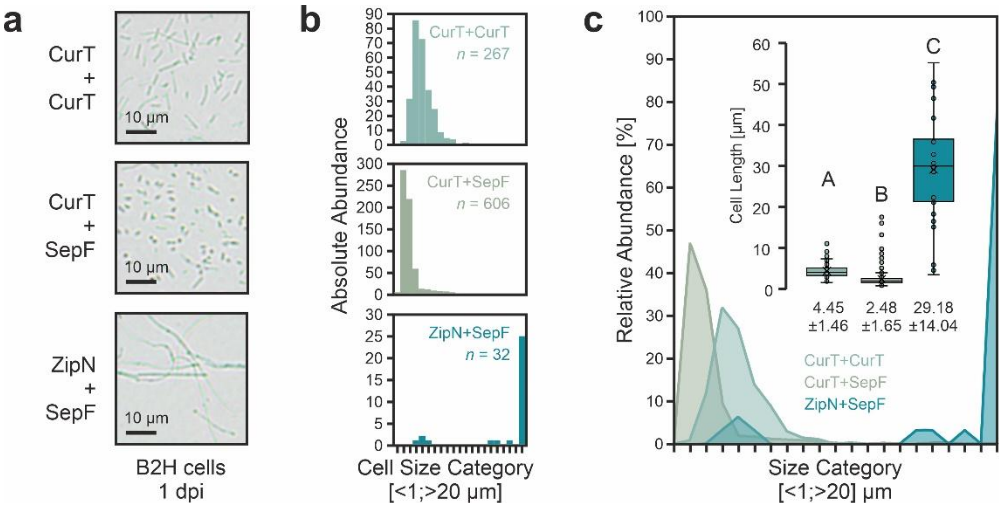

Supplementary Fig. 6: BTH101 cell lengths are differentially affected by co-expressing CurT, SepF, and ZipN. a, Microscopic cell size phenotypes of selected BTH101 co-transformants shown in (**Fig. 2a**) 1 day past inoculation (dpi). **b,** Cell length distributions of samples shown in (**a**) with sample sizes (*n*) being indicated. Histograms depict absolute numbers of observations. **c,** Cell length distributions of samples shown in (**a**). Graphs depict relative observation distributions, and inset boxplots depict precise data point distributions. Boxplot centre line = median; cross = mean; boxes = 25th‑75th percentiles; whiskers = 1.5 × IQR; circles = outliers; all datapoints shown. Values below plots indicate mean ± SD. Precise sample sizes (number of measured cells *n*) are given in panel (**b**). Numbers below boxplots correspond to average values ± standard deviation. Capital letters indicate statistically significant differences according to multiple simultaneous comparisons in *post hoc* Bonferroni–Holm-corrected Tukey HSD (honest significant difference) tests after significant among-group differences were detected by two-sided one-way ANOVA ($p = 1.11 \times 10^{-16}$). The experiment was performed with two independently co-transformed BTH101 clones yielding similar results.

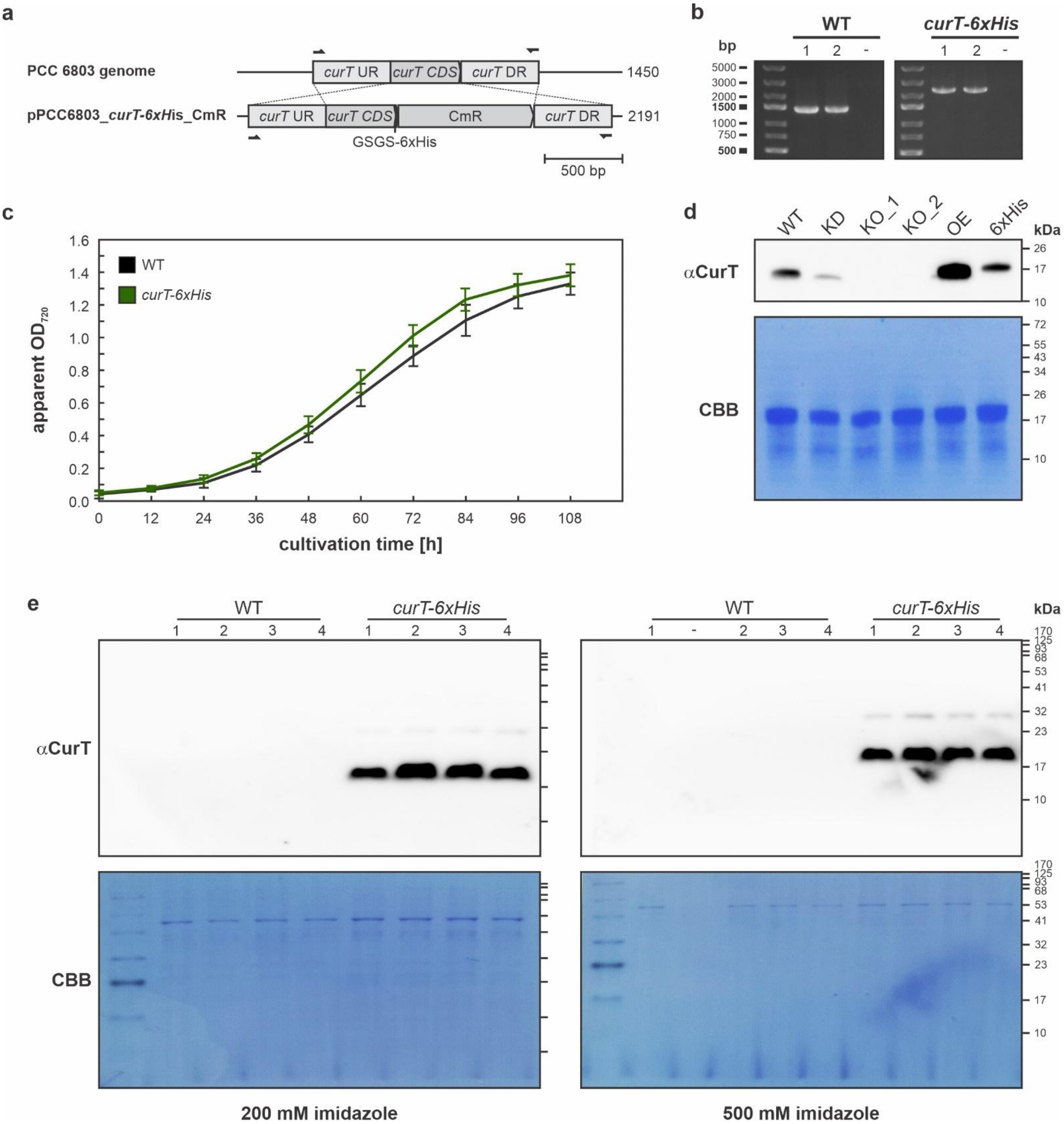

Supplementary Fig. 7: Ni-NTA pulldown of *Synechocystis* CurT-associated proteins *via* C-terminally 6xhistidine-tagged CurT. a, Schematic maps of the genomic *curT* locus and the pPCC6803_*curT*-6xHis_CmR construct used in this study. CmR, chloramphenicol resistance gene *cat*; UR, upstream (5') region; DR, downstream (3') region; CDS, coding sequence. UR/DR regions used for homologous recombination with genomic *curT* (*slr0483*) target locus are indicated as grey boxes. Primer-binding sites for genotyping PCR are indicated as half arrows. Expected amplicon sizes are indicated in units of base pairs. Constructs are drawn to scale according to scale bar. **b,** Genotyping PCR confirming complete replacement of all chromosomal *curT* gene copies of WT cells through the *curT-6xHis*_CmR construct in two independent strains of *curT-6xHis* cells. **c**, Growth curves of WT and *curT-6xHis* strains cultivated for 108 h at 25 °C and 50 µmol photons m^{-2} s^{-1} recorded as optical density (OD) at wavelength λ = 720 nm (apparent OD_{720}; PSI Multicultivator inbuilt photometer). Growth data was collected simultaneously to data depicted in Fig. 1d. Data points represent means and error bars represent standard deviation for *n* = 4 biological replicates.

d, Immunoblot analysis of whole-cell protein extract confirming expected size shift and cellular CurT-6xHis protein accumulation similar to WT levels. Coomassie brilliant blue (CBB) staining of the PVDF blotting membrane is provided as a loading control. WT, wild type; KD, knock-down; KO, knock-out; OE, overexpression. **e**, Immunoblot analysis of Ni-NTA bound protein fractions after elution with 200 (left) and 500 (right) mM imidazole confirming successful enrichment of CurT-6xHis in four independent experiments. CBB staining of the PVDF blotting membrane is provided as a loading control. The Ni-NTA pulldown experiment and subsequent proteomic analysis was performed twice independently on *n* = 4 biological replicates of WT and *curT-6xHis* with similar results.

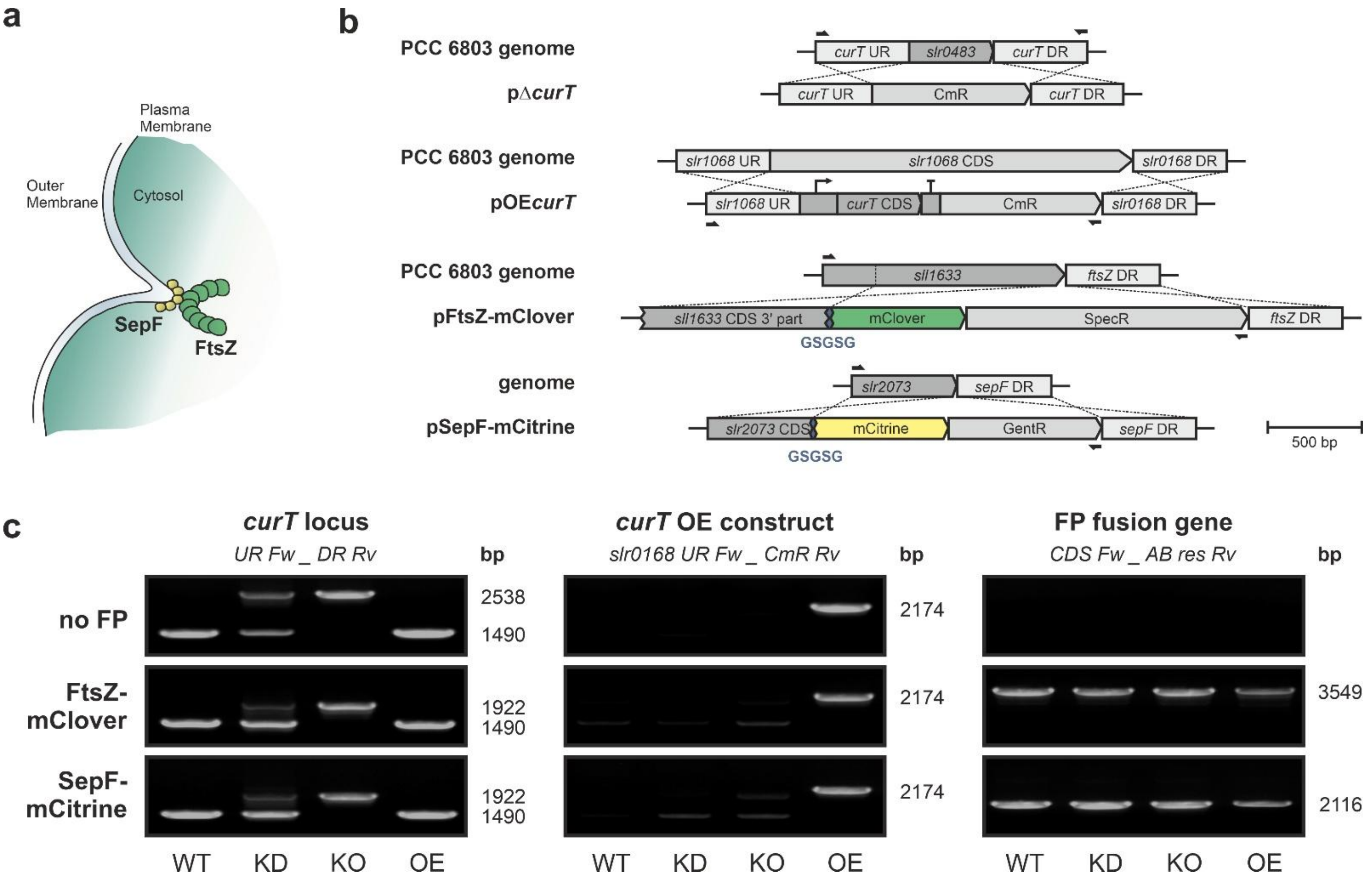

Supplementary Fig. 8: Introduction of genetic constructs encoding fluorescent-protein tagged versions of *Synechocystis* FtsZ (mClover) and SepF (mCitrine) into WT and *curT* mutant strains through homologous recombination. a, Schematic overview if the localizations of FtsZ (green) and SepF (yellow) during undisturbed cell division in *Synechocystis* sp. PCC 6803. **b,** Schematic maps of the pΔcurT_CmR knock-out and the pOEcurT_CmR overexpression constructs, as well as *ftsZ-mClover* and *sepF-mCitrine* fluorescent-protein gene fusion constructs used in this study. CmR, chloramphenicol resistance gene *cat*; SpecR, spectinomycin resistance gene *aadA*; GentR, gentamycin resistance gene *aacC1*; UR, upstream (5') region; DR, downstream (3') region; CDS, coding sequence. UR/DR regions used for homologous recombination with genomic *curT* (*slr0483*), neutral site (*slr0168*), *ftsZ* (*sll1633*), and *sepF* (*slr2073*) target loci are indicated as grey boxes. Primer-binding sites for genotyping PCR are indicated as half arrows. Constructs are drawn to scale according to scale bar in units of base pairs (bp). **c,** Genotyping PCR confirming partial and complete replacement of all chromosomal *curT* gene copies in KD and KO cells, as well as the insertion of an additional *curT* gene copy in the *slr0168* locus for *curT* OE strains, and the insertion of the mClover/mCitrine fluorescent protein (FP) fusion gene constructs into the *ftsZ* and *sepF* loci, respectively. WT, wild type.

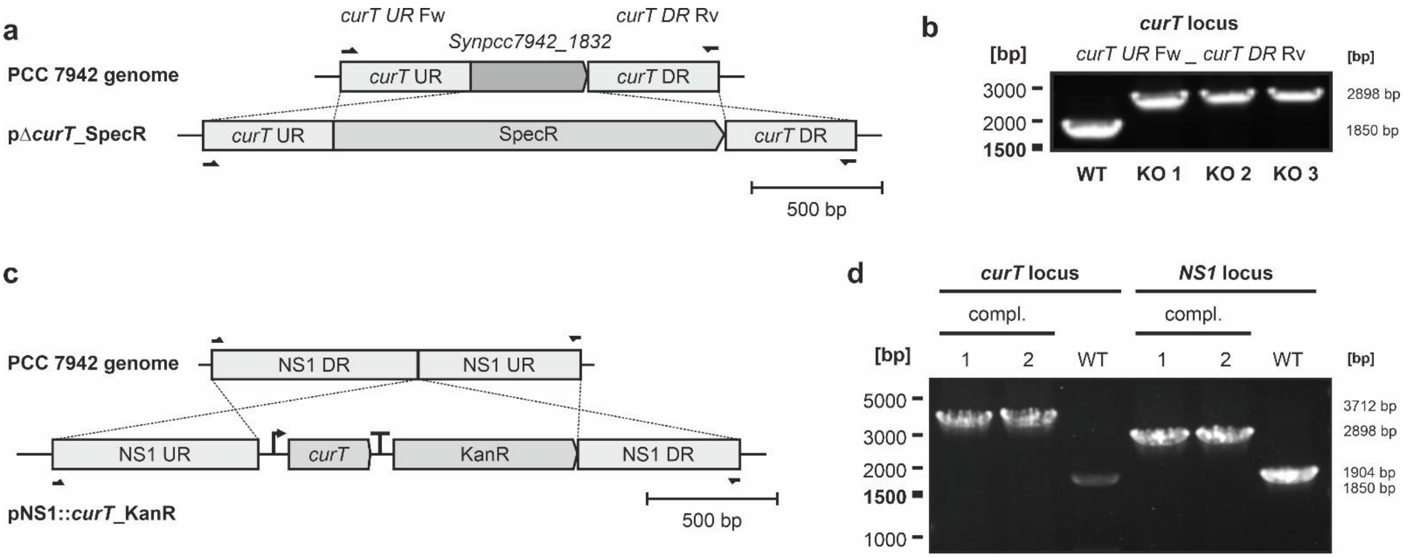

Supplementary Fig. 9: Generation of *Synechococcus curT* KO and complementation mutants. a, Schematic map of the pΔ*curT*_SpecR knock-out construct used in this study. SpecR, spectinomycin resistance gene *aadA*; UR, upstream (5') region; DR, downstream (3') region. UR/DR regions used for homologous recombination with the target genomic *curT* locus (*Synpcc7942_1832*) are indicated as grey boxes. Primer-binding sites for genotyping PCR are indicated as half arrows. The construct is drawn to scale according to scale bar in units of base pairs (bp). **b,** The *curT* coding sequence (*Synpcc7942_1832*) has been replaced with a SpecR resistance cassette by homologous recombination. Genotyping PCR confirms complete replacement of all chromosomal *curT* gene copies in three independent clones of *Synechococcus curT* KO cells. WT, wild type. **c,** Schematic map of the pNS1::*curT*_KanR complementation construct used in this study. KanR, kanamycin resistance gene *nptI*; UR, upstream (5') region; DR, downstream (3') region. UR/DR regions used for homologous recombination with the target genomic *neutral site* locus NS1 are indicated as grey boxes. Primer-binding sites for genotyping PCR are indicated as half arrows. The construct is drawn to scale according to scale bar in units of base pairs (bp). **d**, In *curT* complementation (compl.) strains the endogenous *curT* coding sequence has been replaced with a SpecR resistance cassette and the NS1 locus has been replaced by a copy of the curT gene (200 bp UR, *curT* coding sequence 100 bp DR) by homologous recombination. Genotyping PCR confirms complete replacement of all endogenous chromosomal *curT* gene copies and NS1 loci in two independent clones of *Synechococcus curT* KO cells.

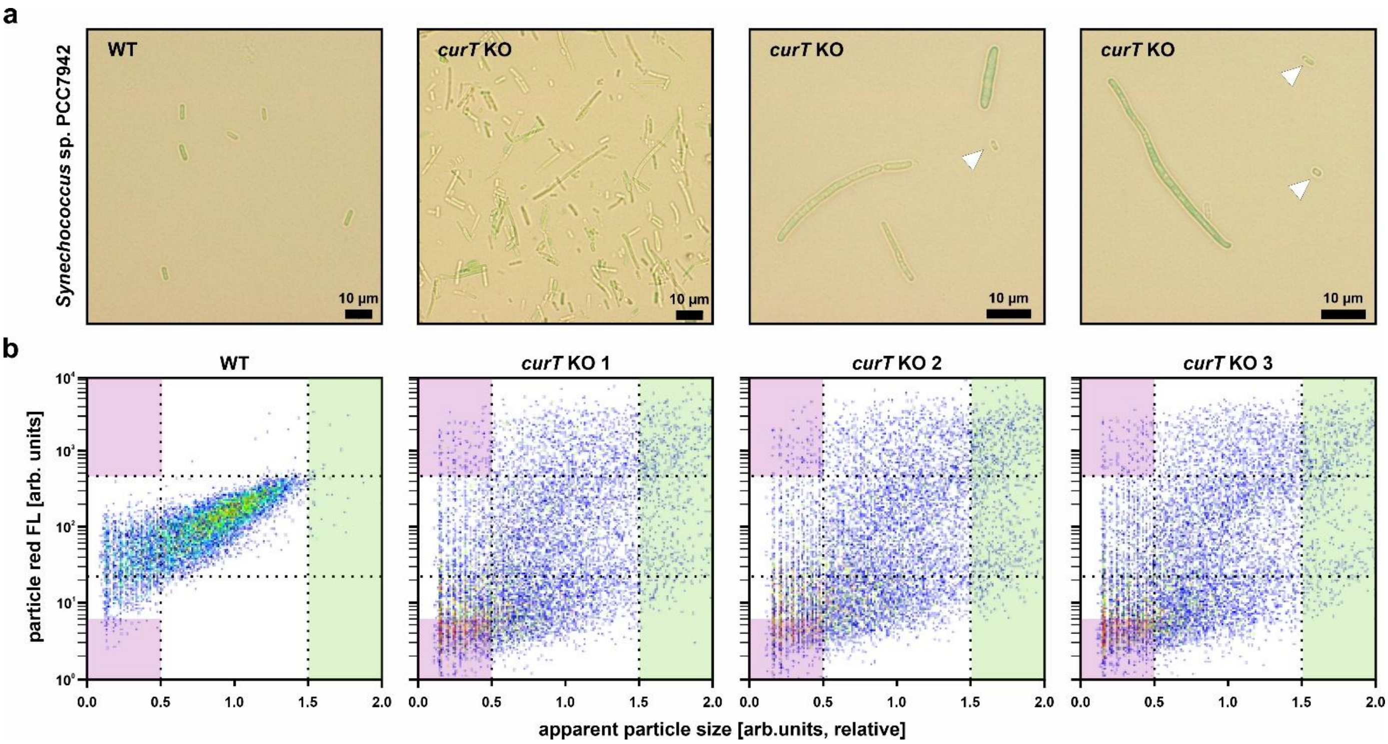

Supplementary Fig. 10: *Synechococcus curT* mutants display asymmetric and impaired cell division. a, Representative bright field micrographs of *Synechococcus* WT and *curT* KO strains. White arrow heads signify *curT* KO mini cells. **b,** Cell-size distributions of *Synechococcus curT* mutants as estimated by particle sizer. Relative particle size (x axis) was estimated from uncalibrated forward scatter (FSC). Red fluorescence (FL) intensity (y axis, arbitrary (arb.) units; excitation 532 nm, emission 680 ± 15 nm) is shown as detector output values without absolute calibration. Dot plots are colored by point density. WT and *n* = 3 independent *curT* KO mutant clones were assayed. Data represents cells taken from solid BG11 growth media grown under continuous illumination. Coloured panels signify size ranges largely absent from the WT control (magenta, mini cell range; green, filamentous cell range).The experiment was performed twice independently with similar results.

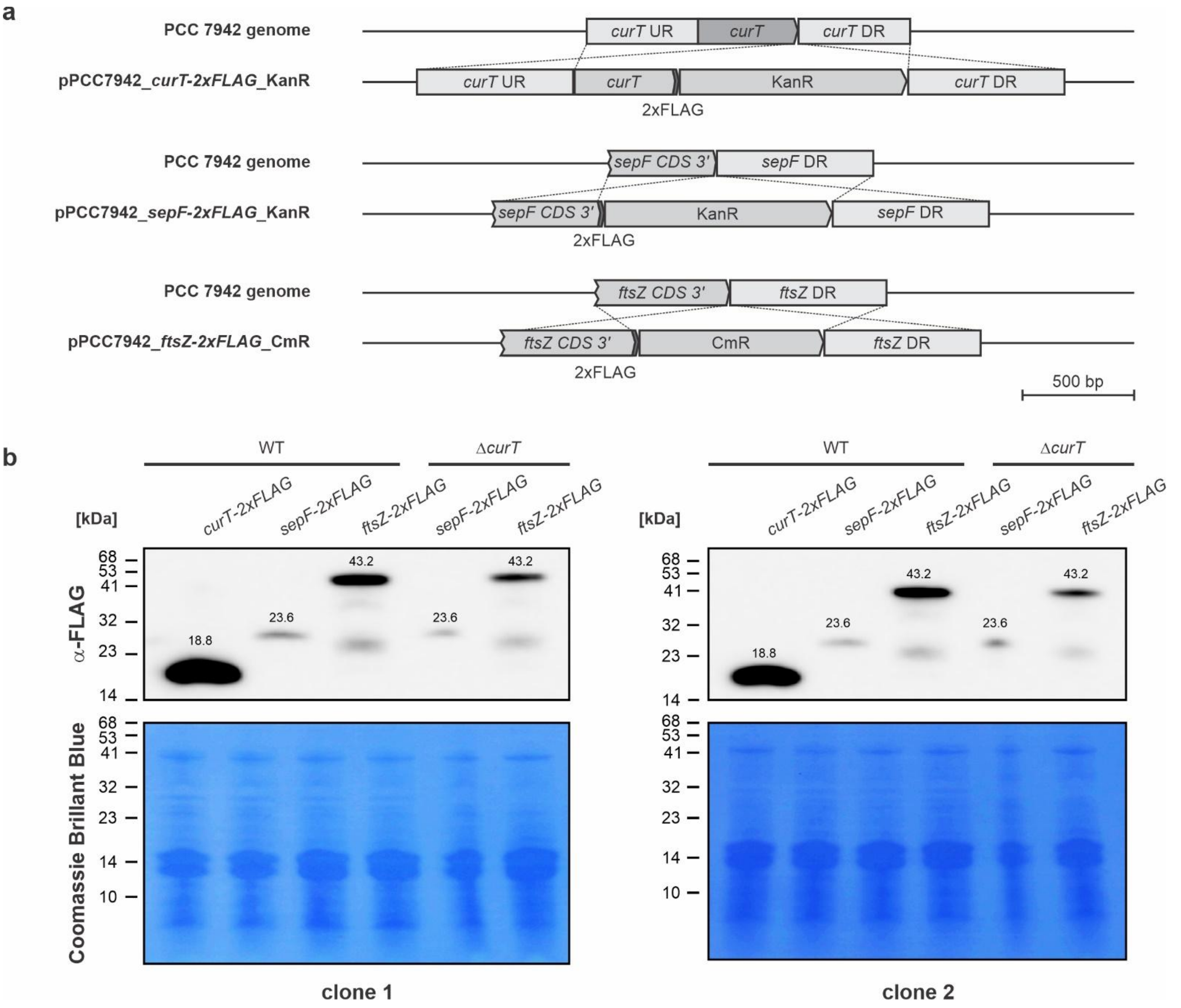

Supplementary Fig. 11: C-terminal 2xFLAG-tagging of *Synechococcus* CurT, SepF, and FtsZ. **a,** Schematic maps of the *Synechococcus elongatus* PCC 7942 genomic *curT* (Synpcc7942_1832)*,* *sepF* (Synpcc7942_2059), and *ftsZ* (Synpcc7942_2378) loci and the corresponding 2xFLAG-tagging constructs used in this study. KanR, kanamycin resistance gene *nptI*: CmR, chloramphenicol resistance gene *cat*; UR, upstream (5’) region; DR, downstream (3’) region; CDS, coding sequence. UR/DR regions used for homologous recombination with genomic target loci are indicated as grey boxes. Constructs are drawn to scale according to scale bar. **b**, Immunoblot analysis of whole-cell protein extract confirming accumulation of CurT-2xFLAG, SepF-2xFLAG, and FtsZ-2xFLAG in WT and *curT* KO mutant (Δ*curT*) genetic backgrounds. Coomassie brilliant blue staining of the PVDF blotting membranes is provided as a loading control. Expected sizes of the 2xFLAG-tagged proteins are indicated above the corresponding bands. Immunodetection in mutant strains was repeated independently two times with similar results.

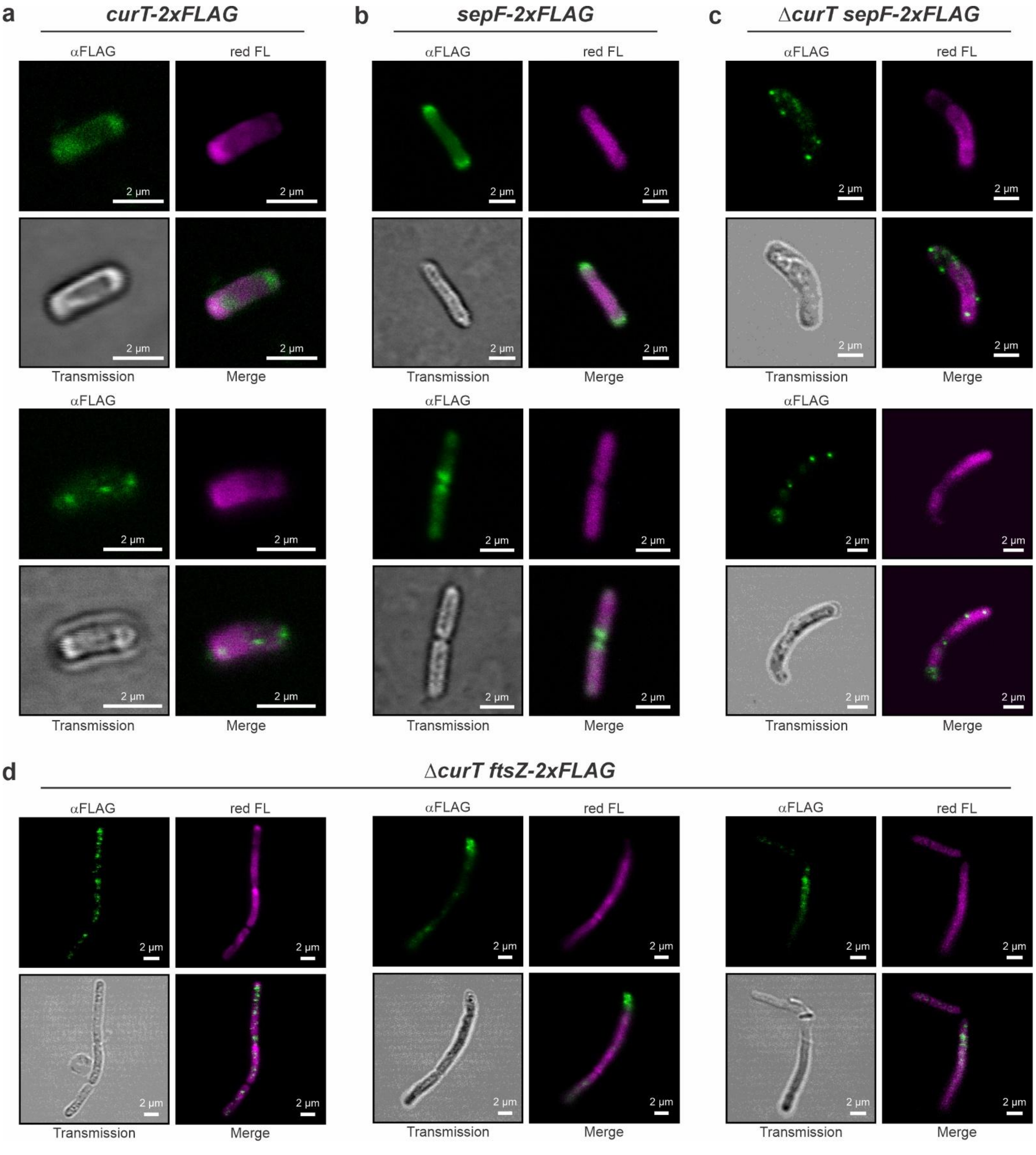

Supplementary Fig. 12: Subcellular localization of *Synechococcus* CurT-2xFLAG, SepF-2xFLAG, and FtsZ-2xFLAG by immunofluorescent staining. Subcellular localization of CurT-2xFLAG in WT background (**a**), SepF-2xFLAG in WT (**c**) and Δ*curT* knock-out mutant background (**c**), and FtsZ-2xFLAG in Δ*curT* knock-out mutant background (**d**) of *Synechococcus* elongatus PCC 7942 by immunofluorescent staining. Primary antibody: anti-FLAG (mouse) (1:100); secondary antibody: Alexa Fluor 488 goat anti-mouse IgG H&L (1:400). Alexa Fluor 488 emission (green) was detected at 500-540 nm; chlorophyll and phycobiliprotein red fluorescence (red FL.; magenta) was detected at 670-750 nm. Immunofluorescent staining experiments were repeated independently at least two times with similar results.

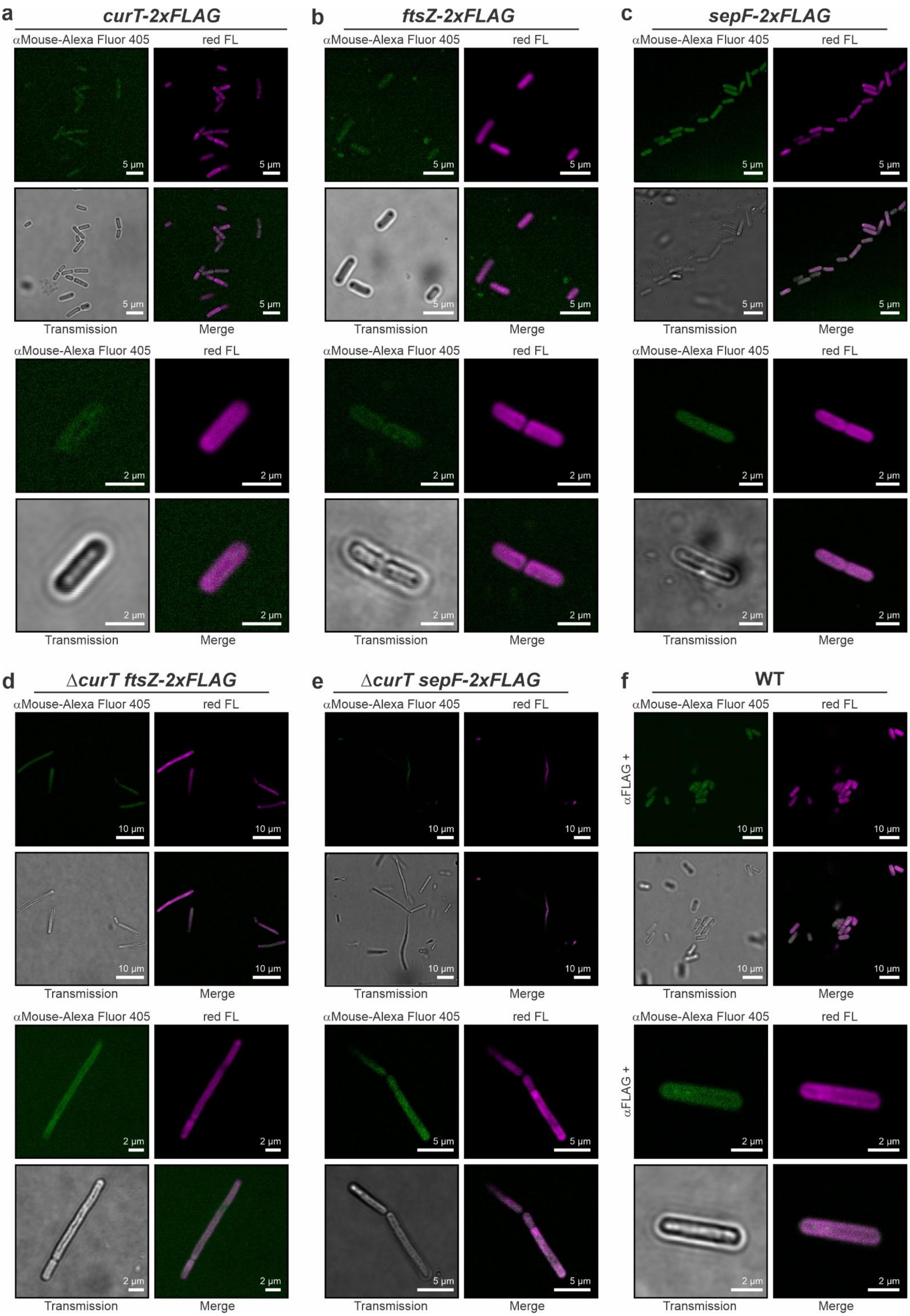

a
curT-2xFLAG
b
ftsZ-2xFLAG
c
sepF-2xFLAG
αMouse-Alexa Fluor 405
red FL
Transmission
Merge
5 µm
2 µm
d
ΔcurT ftsZ-2xFLAG
e
ΔcurT sepF-2xFLAG
f
WT
αFLAG +
10 µm

Supplementary Fig. 13: Negative controls of *Synechococcus* CurT-2xFLAG, SepF-2xFLAG, and FtsZ-2xFLAG immunofluorescent staining. Cells were stained with secondary antibody Alexa Fluor 488 goat anti-mouse IgG H&L (1:400). Alexa Fluor 488 emission (green) was detected at 415-500 nm; chlorophyll *a* and phycobiliprotein red fluorescence (red FL; magenta) was detected at 670-750 nm. Strains expressing CurT-2xFLAG (**a**), FtsZ-2xFLAG (**b**), and SepF-2xFLAG (**c**) in the WT genetic background, as well as FtsZ-2xFLAG (**d**), and SepF-2xFLAG (**e**) in the *Synechococcus curT* deletion mutant genetic background are shown. **f**, WT cells stained with primary antibody anti-FLAG (mouse) (1:100) and secondary antibody Alexa Fluor 488 goat anti-mouse IgG H&L (1:400). Immunofluorescent staining experiments were repeated independently two times with similar results.

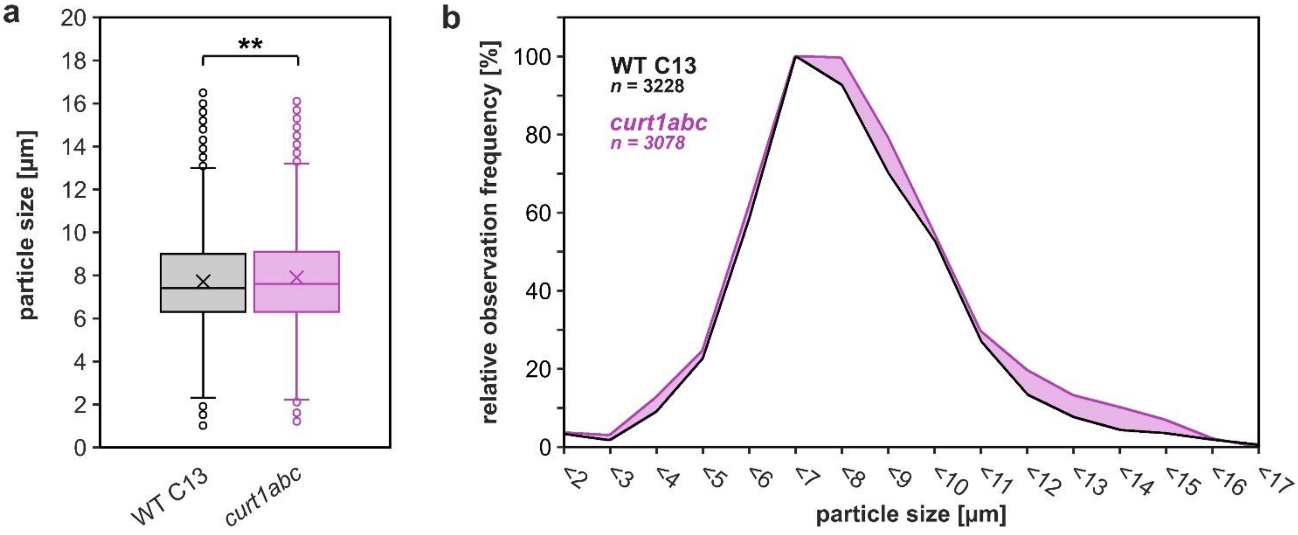

Supplementary Fig. 14: *Chlamydomonas curt1abc* mutant cells are larger than WT C13. a, Cell-size estimate distributions of *Chlamydomonas* WT C13 and *curt1abc* mutants. Boxplot horizontal lines represent the median, crosses represent average values, and boxes indicate the 25th and 75th percentiles. Whiskers extend 1.5-fold the interquartile range with outliers being represented as circles beyond the range of the whiskers. Asterisks indicate statistically significant difference according to two-sided, unpaired, heteroscedastic *t*-test (p = 0.0015). **b,** Cell size distribution with observation frequency normalized to maximum relative abundance. Light magenta areas highlight relative overabundance of smaller and larger cells in *curt1abc* as compared to WT C13. Graphs represent data from exponential growth phase cultures grown in TAP media under continuous illumination. Sample sizes *n* (*i.e.*, numbers of cell observed) are indicated in (**b**). The experiment was repeated independently two times with similar results.

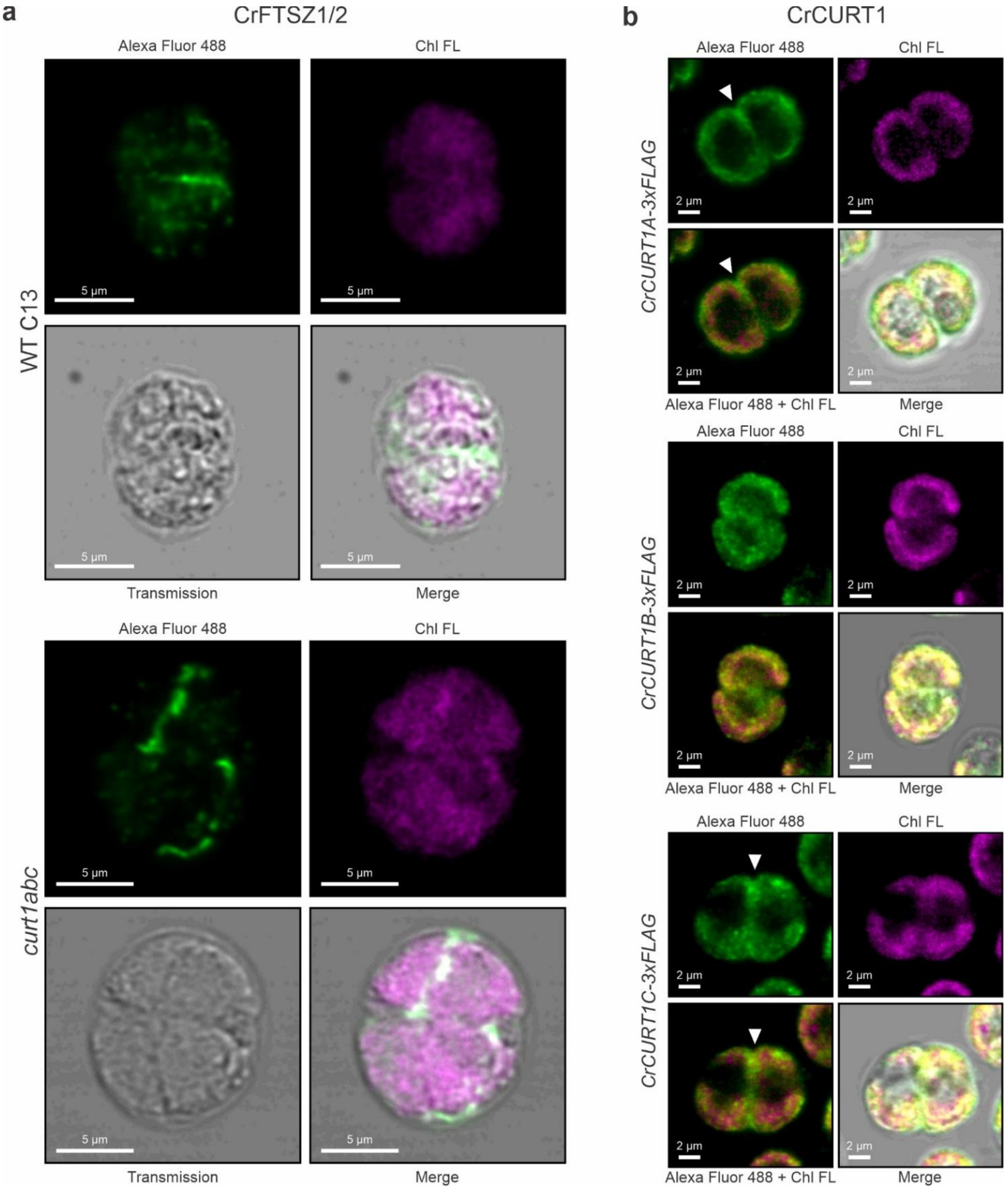

Supplementary Fig. 15: *Chlamydomonas CURT1A*, *B*, and *C* affect chloroplast FTSZ-ring formation and differentially localize to the plastid division site. a, Chloroplast FTSZ ring formation in WT an *curt1abc* mutant strains as visualized by immunofluorescent staining with anti-*Arabidopsis thaliana* FTSZ2 antibody. **b**, Subcellular localization of CrCURT1A-3xFLAG (top), CrCURT1B-3xFLAG (middle), and CrCURT1C-3xFLAG (bottom) expressed in C13 WT cells by immunofluorescent staining. Cells were cultured in a 12h light / 12h dark cycle in TAP media and sampled 20 minutes after light-to-dark-transition. Primary antibody: **a**, anti-AtFtsZ2 (rabbit); **b,** anti-FLAG (mouse) (1:500); secondary antibody: **a,** Alexa Fluor 488 goat anti-Rabbit IgG H&L (1:500); **b,** Alexa Fluor 488 goat anti-mouse IgG H&L (1:500). Alexa Fluor 488 emission (green) was detected at 500-540 nm; chlorophyll fluorescence (Chl FL; magenta) was detected at 670-750 nm. White arrowheads indicate CrCURT1A-3xFLAG and CrCURT1C-3xFLAG accumulation in the chloroplast division plane. Yellow indicated co-localization of Chl FL and Alexa Fluor 488 emission signals. Immunofluorescent staining experiments were repeated independently at least two times with similar results.

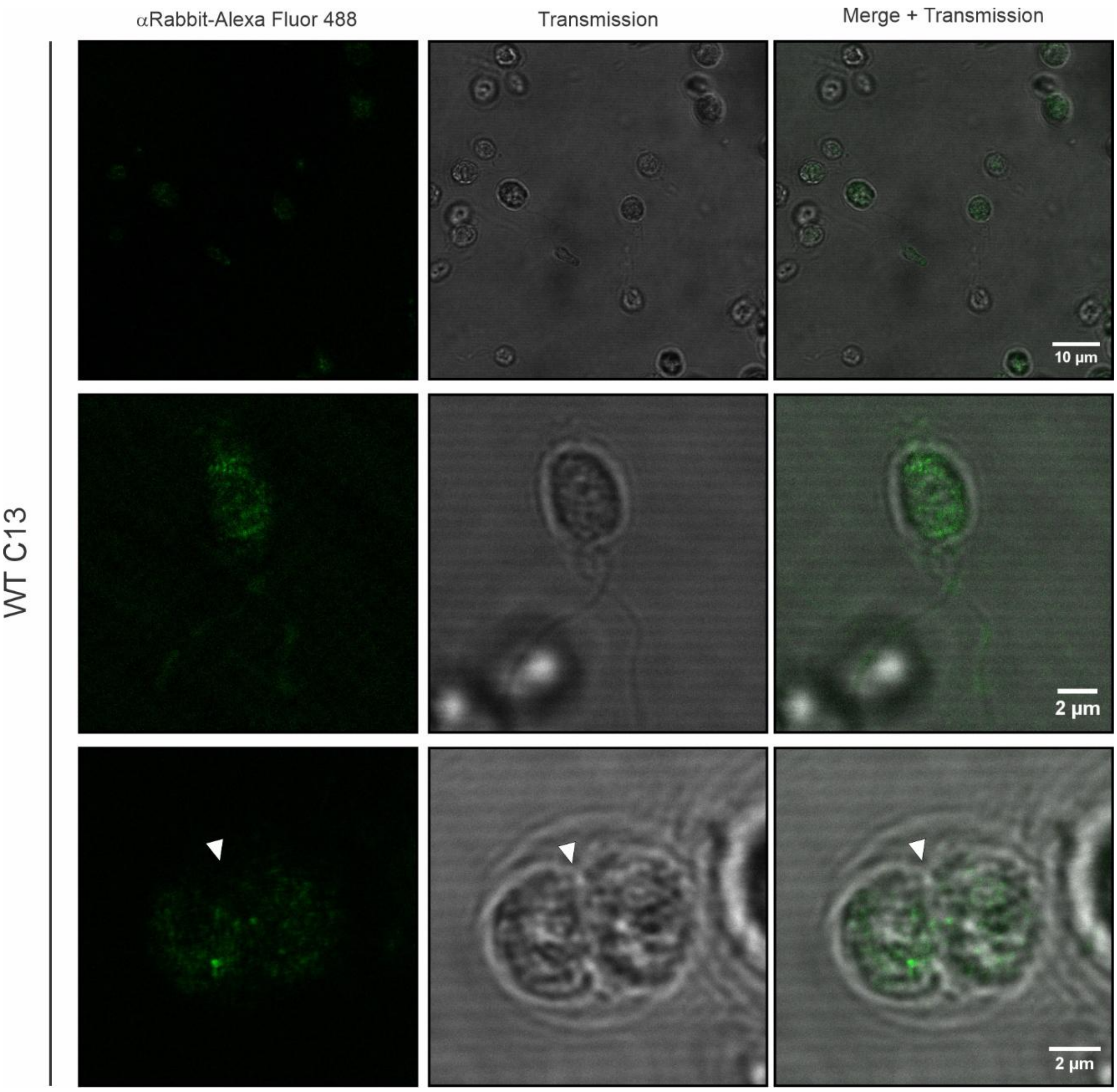

Supplementary Fig. 16: Immunofluorescent staining negative control of *Chlamydomonas* WT cells. Exponential growth phase C13 WT cells were stained with secondary antibody Alexa Fluor 488 goat anti-rabbit IgG H&L (1:500). Alexa Fluor 488 emission was detected at 500-540 nm. White arrowheads indicate the chloroplast division plane. Immunofluorescent staining experiments were repeated independently two times with similar results.

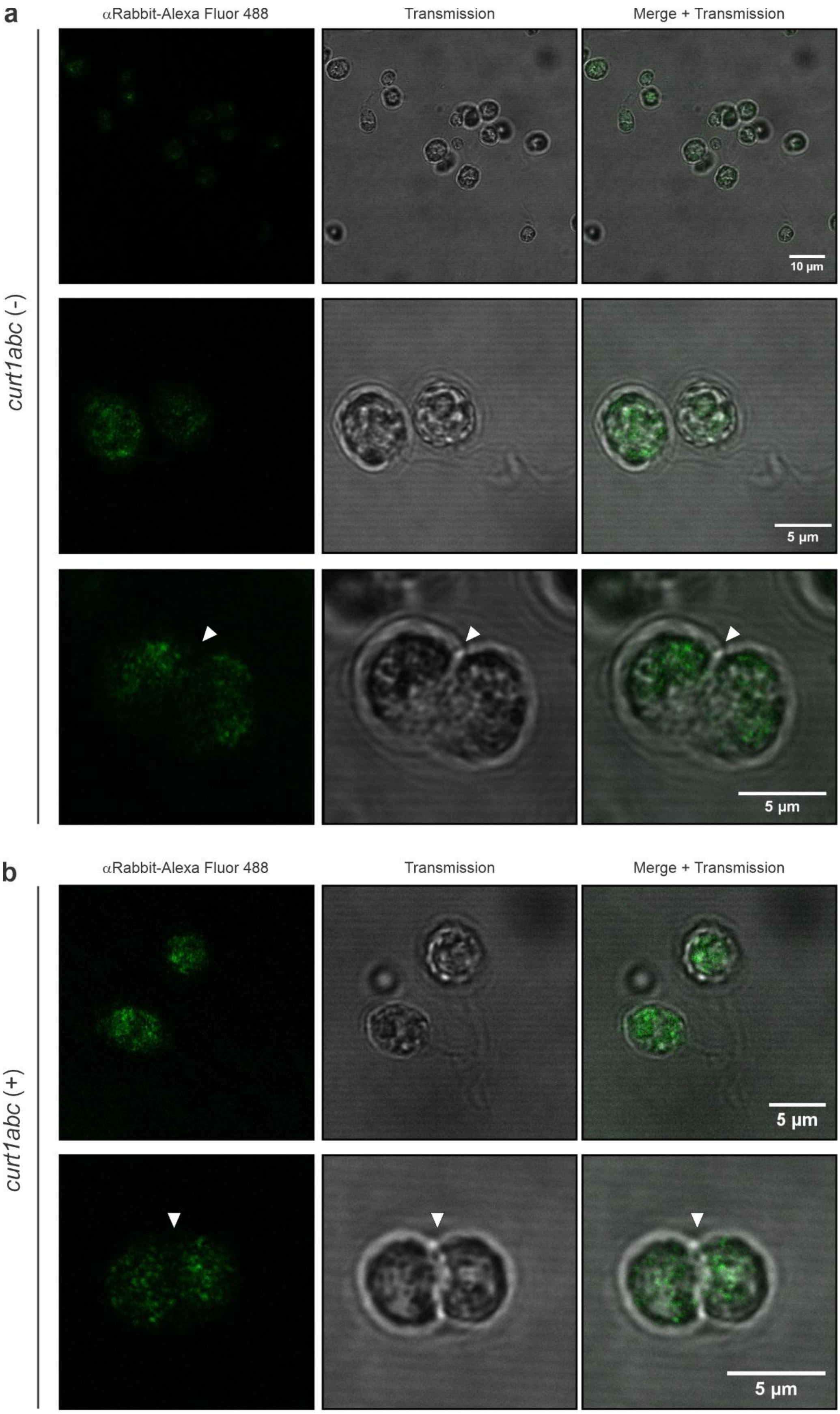

Supplementary Fig. 17: Immunofluorescent staining negative control of *Chlamydomonas curt1abc* mutant cells. Exponential growth phase *curt1abc* cells were stained with secondary antibody Alexa Fluor 488 goat anti-Rabbit IgG H&L (1:500). Alexa Fluor 488 emission was detected at 500-540 nm. White arrowheads indicate the chloroplast division plane. **a**, *curt1abc* mating type (-). **b**, *curt1abc* mating type (+). Immunofluorescent staining experiments were repeated independently two times with similar results.

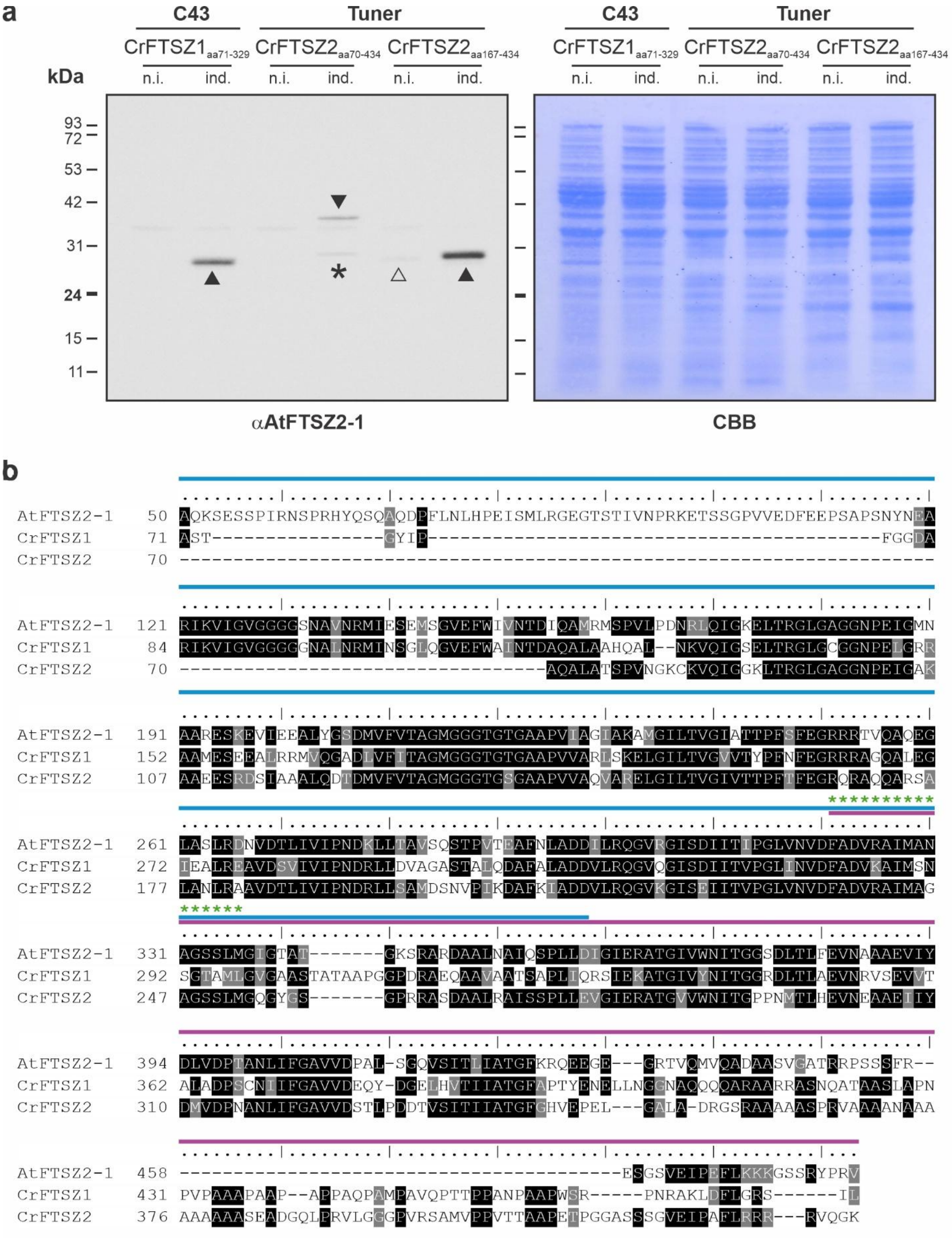

Supplementary Figure 18. The anti-AtFTSZ2 antibody used in *Chlamydomonas* IF studies recognizes CrFTSZ1 and CrFTSZ2. a, Immunoblot analysis of recombinant 6xHis-CrFTSZ1 N-terminal fragment (aa 71-435; expected size 29.1 kDa) expressed in *E. coli* C43 (left), full-length mature recombinant CrFTSZ2-8xHis (aa 70-435; expected size 36.8 kDa) (middle), and recombinant CrFTSZ2-8xHis C-terminal fragment (aa 167-435; expected size 29.0 kDa) expressed in *E. coli* Tuner^{TM} (DE3) cells. Whole-cell extracts corresponding to OD_{600} = 0.2 cell equivalents were probed in non-IPTG-induced (n.i.) and IPTG-induced (ind.) states, respectively. kDa, kilo-Dalton. Coomassie brilliant blue (CBB) staining of the PVDF membrane is provided as loading control. Specific signals are indicated by black arrowheads (empty arrow head: leaky expression). A tentative CrFTSZ2 degradation product is indicated by an asterisk. Induction and subsequent

immunodetection was repeated independently two times with similar results. **b**, Protein sequence alignment of mature AtFTSZ2-1, CrFTSZ1, and CrFTSZ2 visualized by boxshade. Identical (black) and similar (grey) residues are highlighted. A highly immunogenic amino-acid stretch of AtFTSZ2-1 (indicated by green asterisks below the amino-acid sequence alignment) used in previous immunization studies reveals higher local similarity of CrFTSZ1 than CrFTSZ2 to AtFTSZ2-1, corroborating reactivity of anti-AtFTSZ2 to CrFTSZ1 and CrFTSZ2. Subsequences corresponding to N-terminal CrFTSZ1 (blue) and C-terminal CrFTSZ2 (magenta) fragments are indicated above the amino-acid alignment.

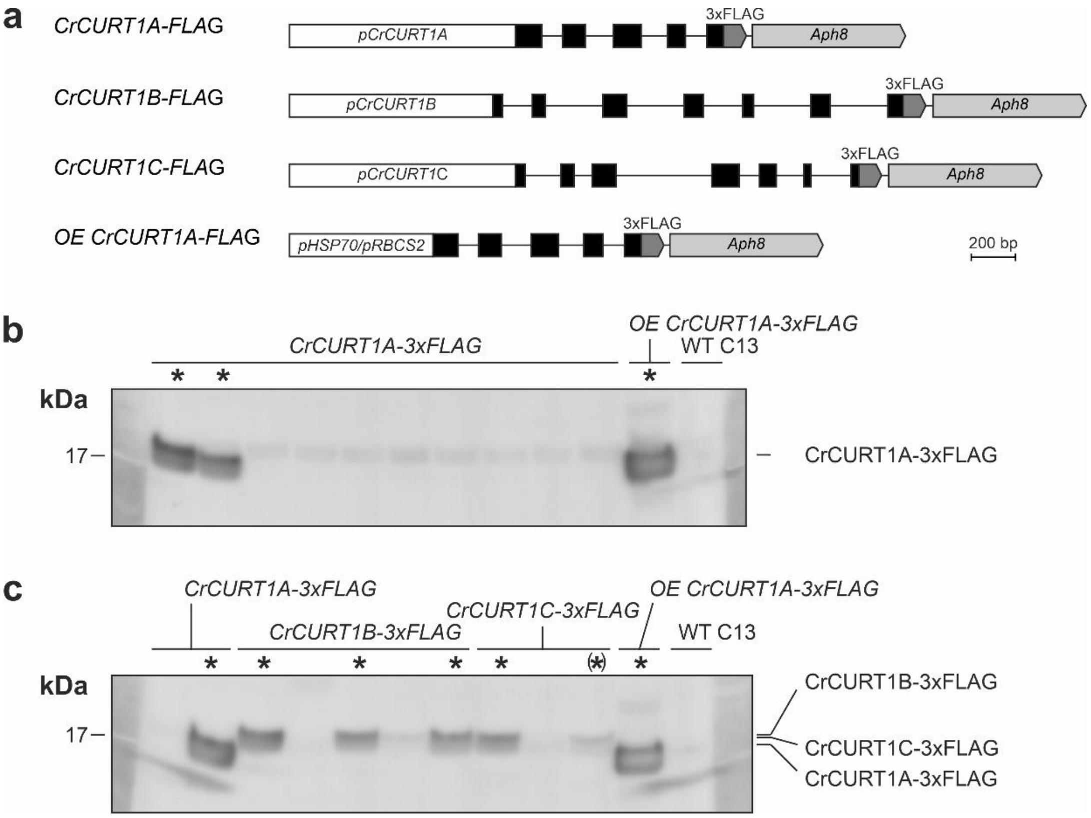

Supplementary Fig. 19: *Chlamydomonas reinhardtii CURT1A/B/C-3xFLAG* expression constructs and *curt1abc* complementation strain immunoblot analysis. a, Schematic maps of CrCURT1A/B/C-3xFLAG native-level expression and CrCURT1A-3xFLAG over-expression (OE) constructs used for *curt1abc* mutant complementation (top to bottom). Black boxes indicate exons of native gene structures. *pCrCURT1A/B/C* indicate native promoter regions (genomic upstream regions of respective first exon). *Aph8*, paromomycin resistance cassette. Scale bar in units of base pairs (bp). **b,** Immunoblot analysis of C-terminally 3xFLAG-fused CURT1A protein. **c,** Immunoblot analysis of C-terminally 3xFLAG-fused CURT1A, CURT1B, and CURT1C proteins. OE CURT1A-3xFLAG, a clone expressing CURT1A-3xFLAG under the control of the *pHSP70A*/*pRBCS2* tandem promoter was used as a positive control and C13, a wild-type strain, was used as a negative control. Whole cell extracts corresponding to 2 mg of chlorophyll were loaded on each lane. Asterisks indicate clones suitable for immunofluorescent staining of FLAG-tagged CURT1 protein. kDa, kilo-Dalton (apparent molecular weight marker). Immunodetection was repeated independently two times with similar results.

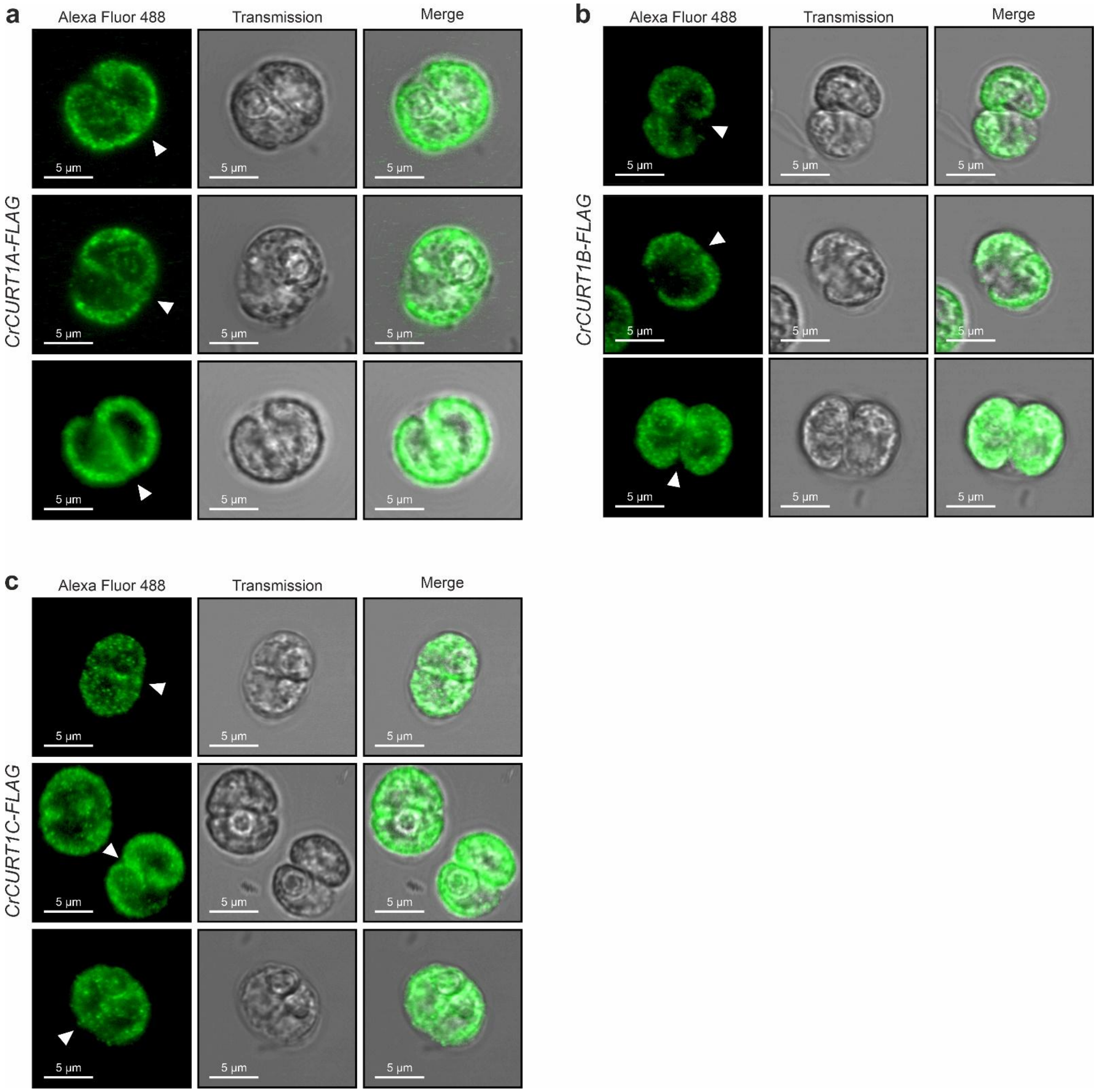

Supplementary Fig. 20: Subcellular localization of CrCURT1-3xFLAG fusion proteins by immunofluorescent staining of dividing *Chlamydomonas* cells. **a,** CrCURT1A-3xFLAG expressed in C13 WT cells. **b,** CrCURT1B-3xFLAG expressed in C13 WT cells. **c,** CrCURT1C-3xFLAG expressed in C13 WT cells. Cells were cultured in a 12h light / 12h dark cycle in TAP media and sampled 30 minutes after light-to-dark-transition. Primary antibody: anti-FLAG (mouse) (1:500); secondary antibody: Alexa Fluor 488 goat anti-mouse IgG H&L (1:500). Alexa Fluor 488 emission was detected at 500-540 nm; chlorophyll fluorescence (Chl FL) was detected at 670-750 nm. White arrowheads indicate the chloroplast division plane. Duration of fixation of the shown cells in methanol had to be extended as compared to data shown in **Fig. 7** and **Supplementary Fig. 15** in order to achieve sufficient staining intensity. Immunofluorescent staining experiments were repeated independently two times with similar results. Cells shown are representative of *n* = 3/2/3 biological replicates for CrCURT1A/B/C-3xFLAG, respectively.

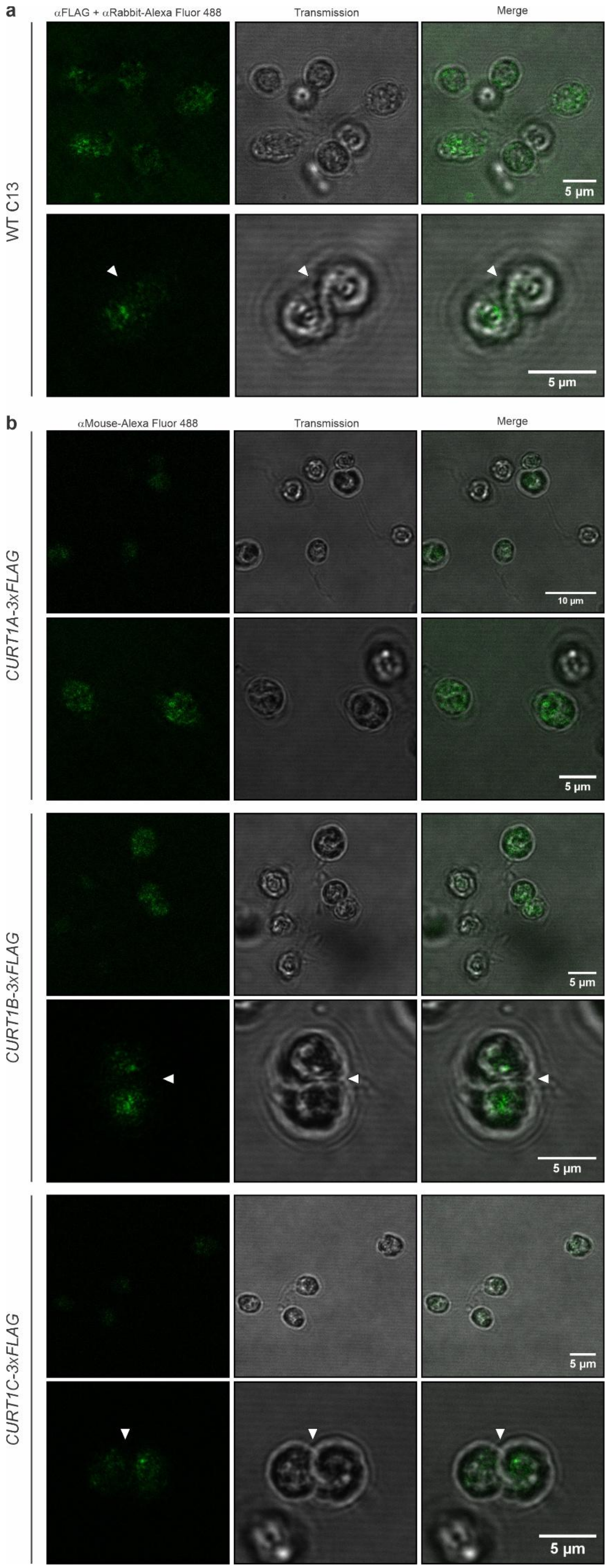
a
αFLAG + αRabbit-Alexa Fluor 488
Transmission
Merge
WT C13
5 µm
5 µm
b
αMouse-Alexa Fluor 488
Transmission
Merge
CURT1A-3xFLAG
10 µm
5 µm
CURT1B-3xFLAG
5 µm
5 µm
CURT1C-3xFLAG
5 µm
5 µm

Supplementary Fig. 21: Immunofluorescent staining WT and negative control of CrCURT1-3xFLAG fusion protein expression strains of *Chlamydomonas*. a, C13 WT cells were stained with primary antibody anti-FLAG (mouse) (1:500) and secondary antibody Alexa Fluor 488 goat anti-mouse IgG H&L (1:500). **b**, CrCURT1A-3xFLAG (top), CrCURT1B-3xFLAG (middle), and CrCURT1B-3xFLAG (bottom) expression strains were stained with secondary antibody Alexa Fluor 488 goat anti-mouse IgG H&L (1:500). Exponential growth phase cells were stained, and Alexa Fluor 488 emission was detected at 500-540 nm. White arrowheads indicate the chloroplast division plane. Immunofluorescent staining experiments were repeated independently two times with similar results.

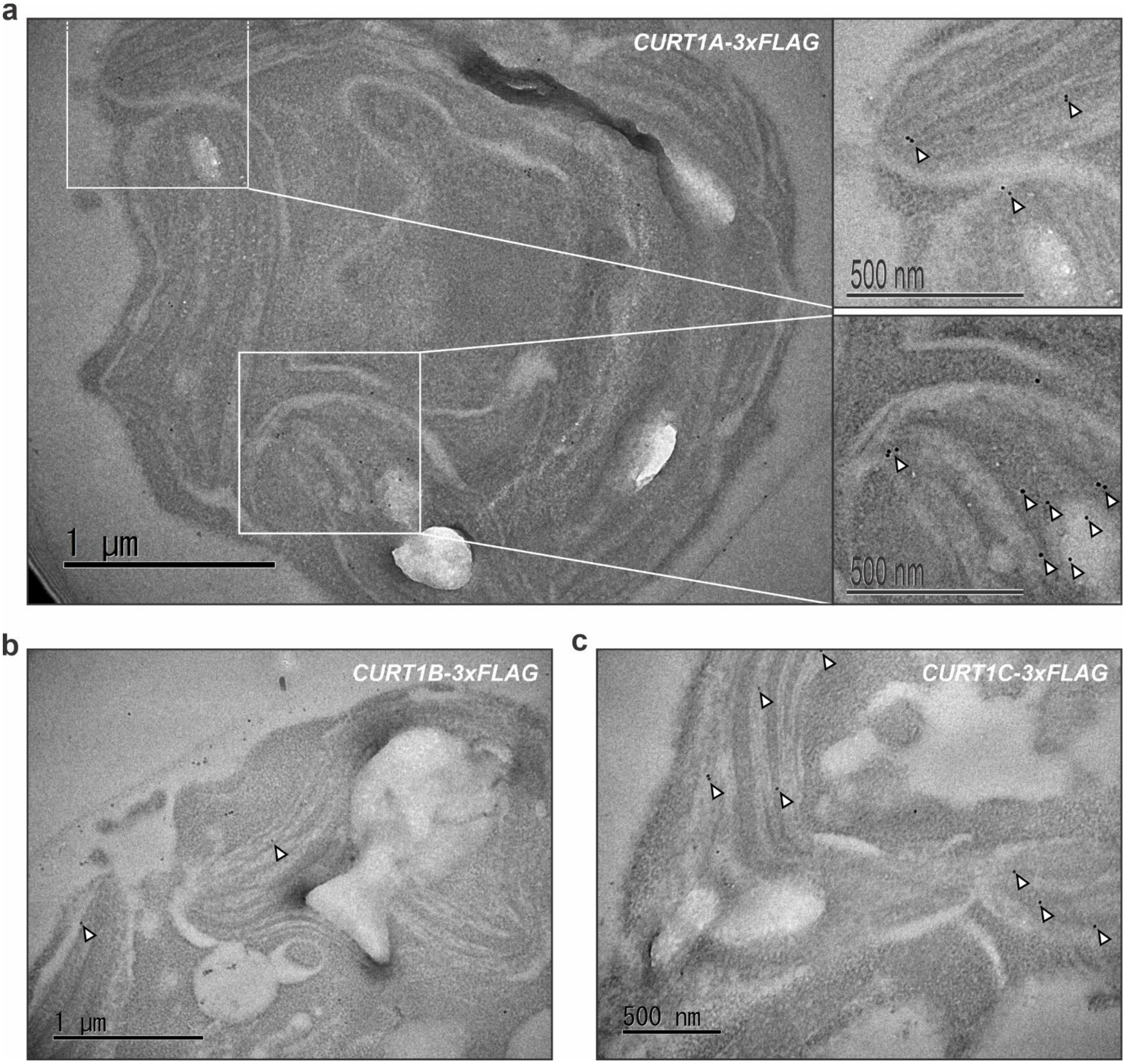

Supplementary Fig. 22: Chloroplast localization of CrCURT1-3xFLAG fusion proteins by immunogold labelling and transmission electron microscopy. **a,** CrCURT1A-3xFLAG expression *curt1abc* complementation cell line. **b,** CrCURT1B-3xFLAG expression *curt1abc* complementation cell line. **C,** CrCURT1C-3xFLAG expression *curt1abc* complementation cell line. Cells were cultured in a 12h light / 12h dark cycle in TAP media and sampled 30 minutes after light-to-dark-transition. Primary antibody: anti-FLAG (mouse; 1:20); secondary antibody: anti-mouse IgG H+L (Gold 10 nm; 1:30). White arrowheads indicate chloroplast-localized gold particles. Localization of CrCURT1A-3xFLAG, CrCURT1B-3xFLAG, and CrCURT1C-3xFLAG is representative of n = 6, n = 7, and n = 6 individual cells obtained from one experiment, respectively. Wildtype cells observed as negative control (n = 4) did not show any specific localization of gold particles (see **Source Data**).
