# Supplementary material for: CurT/CURT1 proteins are involved in cell and chloroplast division coordination of cyanobacteria and green algae
Source: Nat Commun. 2025 Sep 25;16:8424. doi: 10.1038/s41467-025-64163-x (PMC12462475; doi:10.1038/s41467-025-64163-x)
Supplement: Supplementary file 2 — Description of Additional Supplementary Files [file 41467_2025_64163_MOESM2_ESM.pdf]

Description of Additional Supplementary Files

Supplementary Data 1

Primer sequences.

Supplementary Data 2

Chlamydomonas reinhardtii CURT1A/B/C chloroplast transit peptide predictions.

Supplementary Data 3

Synechocystis PCC 6803, Synechococcus PCC 7942 CurT and Chlamydomonas reinhardtii CURT1 protein sequence similarity and identity scores.

Supplementary Data 4

Proteins detected in Chlamydomonas reinhardtii CURT1A-3xFLAG anti-FLAG CoIP experiment (relative to WT control); late-exponential growth phase.

Supplementary Data 5

Proteins detected in Chlamydomonas reinhardtii CURT1B-3xFLAG anti-FLAG CoIP experiment (relative to WT control); late-exponential growth phase.

Supplementary Data 6

Proteins detected in Chlamydomonas reinhardtii CURT1C-3xFLAG anti-FLAG CoIP experiment (relative to WT control); late-exponential growth phase.

Supplementary Data 7

Proteins detected in Chlamydomonas reinhardtii CURT1A-3xFLAG anti-FLAG CoIP experiment (relative to WT control); mid-exponential growth phase.

Supplementary Data 8

Proteins detected in Chlamydomonas reinhardtii CURT1B-3xFLAG anti-FLAG CoIP experiment (relative to WT control); mid-exponential growth phase.

Supplementary Data 9

Proteins detected in Chlamydomonas reinhardtii CURT1C-3xFLAG anti-FLAG CoIP experiment (relative to WT control); mid-exponential growth phase.

Supplementary Data 10

Vector sequences used for genetic engineering of cyanobacteria (annotated, genbank format).

Supplementary Data 11

CrFTSZ expression vector sequences (annotated, genbank format).

Supplementary Data 12

Python custom script for flow cytometry data visualization (density scatter plot).
