# Supplementary material for: CurT/CURT1 proteins are involved in cell and chloroplast division coordination of cyanobacteria and green algae
Source: Nat Commun. 2025 Sep 25;16:8424. doi: 10.1038/s41467-025-64163-x (PMC12462475; doi:10.1038/s41467-025-64163-x)
Supplement: Supplementary file 4 — Reporting Summary [file 41467_2025_64163_MOESM4_ESM.pdf]

nature portfolio

Corresponding author(s): Marcel Dann, Eunchul Kim

Last updated by author(s): Sep 5, 2025

Reporting Summary

Nature Portfolio wishes to improve the reproducibility of the work that we publish. This form provides structure for consistency and transparency in reporting. For further information on Nature Portfolio policies, see our Editorial Policies and the Editorial Policy Checklist.

Statistics

For all statistical analyses, confirm that the following items are present in the figure legend, table legend, main text, or Methods section.

n/a	Confirmed	
[ ]	[x]	The exact sample size (*n*) for each experimental group/condition, given as a discrete number and unit of measurement
[ ]	[x]	A statement on whether measurements were taken from distinct samples or whether the same sample was measured repeatedly
[ ]	[x]	The statistical test(s) used AND whether they are one- or two-sided<br>*Only common tests should be described solely by name; describe more complex techniques in the Methods section.*
[x]	[ ]	A description of all covariates tested
[ ]	[x]	A description of any assumptions or corrections, such as tests of normality and adjustment for multiple comparisons
[ ]	[x]	A full description of the statistical parameters including central tendency (e.g. means) or other basic estimates (e.g. regression coefficient) AND variation (e.g. standard deviation) or associated estimates of uncertainty (e.g. confidence intervals)
[ ]	[x]	For null hypothesis testing, the test statistic (e.g. *F*, *t*, *r*) with confidence intervals, effect sizes, degrees of freedom and *P* value noted<br>*Give P values as exact values whenever suitable.*
[x]	[ ]	For Bayesian analysis, information on the choice of priors and Markov chain Monte Carlo settings
[x]	[ ]	For hierarchical and complex designs, identification of the appropriate level for tests and full reporting of outcomes
[x]	[ ]	Estimates of effect sizes (e.g. Cohen's *d*, Pearson's *r*), indicating how they were calculated

Our web collection on statistics for biologists contains articles on many of the points above.

Software and code

Policy information about availability of computer code

Data collection	Confocal microscopic data was collected using Leica Application Suite X (LAS X) Software

Data analysis	For the analysis of thylakoid volume (Fig. 4), we applied Gaussian blur smoothing using the Leica Application Suite X (LAS X) Software, followed by volume quantification via Fiji software suite (Image Processing Package Fiji (ImageJ2 2.14.0), Schindelin et al., 2012 doi: 10.1038/nmeth.2019), incorporating a "3D Object Counter" plugin.<br><br>Flow cytometry data were converted to text files using the data conversion tool available at https://floreada.io. Flow cytometry data (cell size and red fluorescence) were then processed in Python (v.3.11) using pandas, NumPy, SciPy, and Matplotlib. Two-dimensional scatter density plots were generated with mpl-scatter-density (https://github.com/astrofrog/mpl-scatter-density), applying Gaussian kernel density estimation to visualize event density. Cell size (µm) and red fluorescence (arbitrary units) were plotted on the x- and y-axes, respectively, and figures were exported as high-resolution images (600 dpi). The custom script used is provided in Supplementary Data 12.<br><br>ECL signal quantificatin was performed using ImageJ.<br><br>Morphological comparisons between cyanobacterial wild-type and mutant strain cells were visualized using the ShapePlots tool implemented in the Fiji 57 MicrobeJ 65 plugin, tracing individual cell outlines on bright-field micrographs.<br><br>Statistical analyses were performed using Microsoft Office Excel and the one-way ANOVA with post-hoc test tool as implemented by Navendu Vasavada (https://astatsa.com/).

For manuscripts utilizing custom algorithms or software that are central to the research but not yet described in published literature, software must be made available to editors and reviewers. We strongly encourage code deposition in a community repository (e.g. GitHub). See the Nature Portfolio guidelines for submitting code & software for further information.

Data

Policy information about availability of data

All manuscripts must include a data availability statement. This statement should provide the following information, where applicable:

- Accession codes, unique identifiers, or web links for publicly available datasets
- A description of any restrictions on data availability
- For clinical datasets or third party data, please ensure that the statement adheres to our policy

Proteomics data is annotated and deposited on the DataPlant platform (https://www.nfdi4plants.org/) (Accession Code XXX). Source Data are provided with this paper.

Research involving human participants, their data, or biological material

Policy information about studies with human participants or human data. See also policy information about sex, gender (identity/presentation), and sexual orientation and race, ethnicity and racism.

Reporting on sex and gender	n.a.
Reporting on race, ethnicity, or other socially relevant groupings	n.a.
Population characteristics	n.a.
Recruitment	n.a.
Ethics oversight	n.a.

Note that full information on the approval of the study protocol must also be provided in the manuscript.

Field-specific reporting

Please select the one below that is the best fit for your research. If you are not sure, read the appropriate sections before making your selection.

☒ Life sciences ☐ Behavioural & social sciences ☐ Ecological, evolutionary & environmental sciences

For a reference copy of the document with all sections, see nature.com/documents/nr-reporting-summary-flat.pdf

Life sciences study design

All studies must disclose on these points even when the disclosure is negative.

Sample size	Sample sizes were not predetermined by statistical methods but chosen based on common practice in cyanobacterial and algal cell biology. For each cyanobacterial and algal mutant, a minimum of two independently generated strains was investigated. For growth curves, n = 4 independent biological replicates per strain were used. For quantification of immunoblots, n = 8 biological replicates were quantified. Microscopy panels are representative of n = 2 independent biological replicates, each experiment repeated at least twice with similar results with the exception of transmission electron microscopy, where samples were imaged wthin one experimental run. Cell length and division analyses included ≥60 individual cells per strain. These sample sizes were sufficient to detect robust and statistically significant differences between groups.

Data exclusions	No data were excluded from the analyses. All collected samples, replicates, and cells meeting the defined experimental criteria were included.
Replication	All physiological, molecular biology and biochemical experiments were independently repeated at least twice with consistent results. Growth measurements, immunoblot quantifications, and cell size/shape distributions were performed with the indicated number of biological replicates. Representative images were cconfirmed in at least two (confocal microscopy) independent experiments, or chosen from a sufficiently large number of thin sections (TEM).
Randomization	No formal randomization was applied to sample allocation, as all cultures derived from clonally propagated laboratory strains under identical growth conditions. Data collection and analysis were not influenced by knowledge of sample identity, as quantitative assays (e.g., OD measurements, immunoblots, flow cytometry) were conducted using automated instrumentation.
Blinding	No formal blinding was performed. Experiments such as growth measurements, OD readings, immunoblot quantifications, and flow cytometry were conducted using automated instruments that do not depend on investigator judgment. Image acquisition (confocal and TEM) was performed under comparable instrument settings for all strains within a given experiment, and representative images were selected based on predefined criteria (e.g., cell morphology, fluorescence signal quality) independent of sample identity. Therefore, the absence of blinding is unlikely to bias the results.

Reporting for specific materials, systems and methods

We require information from authors about some types of materials, experimental systems and methods used in many studies. Here, indicate whether each material, system or method listed is relevant to your study. If you are not sure if a list item applies to your research, read the appropriate section before selecting a response.

Materials & experimental systems

n/a	Involved in the study	
☐	☒	Antibodies
☒	☐	Eukaryotic cell lines
☒	☐	Palaeontology and archaeology
☒	☐	Animals and other organisms
☒	☐	Clinical data
☒	☐	Dual use research of concern
☐	☒	Plants

Methods

n/a	Involved in the study	
☒	☐	ChIP-seq
☐	☒	Flow cytometry
☒	☐	MRI-based neuroimaging

Antibodies

Antibodies used	PRIMARY:<br>anti Synechocystis sp. PCC 6803 CurT (rabbit; N-terminus; donated by Dario Leister, LMU Munich); dilution 1:5000 (immunoblot)<br>anti-FLAG monoclonal antibody clone M2 (mouse; F180450UG, Sigma-Aldrich); dilution 1:10000 (immunoblot), 1:100-1:500 (IF staining), dilution 1:20 (immunogold)<br>anti-AtFTSZ2-1 (rabbit; aa250-478; provided by Shin-ya Mijagishima, NIG Mishima); dilution 1:5000 (immunoblot) or 1:1000 (IF staining)<br>anti-DYKDDDDK (mouse; binds to Sigma FLAG, clone FG4R) (AS15 2871, Agrisera) 2 µg / sample (CoIP)<br>anti-DYKDDDDK (rabbit; binds to Sigma FLAG, polyclonal) (AS20 4442 , Agrisera); dilution 1:5000 (Immunoblot)<br><br>SECONDARY:<br>sheep anti-mouse HRP-linked F(ab')$_2$ fragment (GE Healthcare, NA9310V, LOT 4629493); dilution 1:10000 (immunoblot)<br>goat anti-rabbit IgG, HRP-linked antibody (Cell Signalling Technology 7074S); dilution: 1:10000 (immunoblot)<br>goat anti-Rabbit IgG (H&L), HRP conjugated (Agrisera, AS09 602); dilution 1:10000 (immunoblot)<br>Alexa Fluor 488 goat anti-mouse IgG H&L (Invitrogen A11001, LOT 2659299); dilution 1:400-1:1000 (IF staining)<br>Alexa Fluor 488 goat anti-rabbit IgG H&L (Invitrogen A11008, LOT 2775963); dilution 1:500-1:1000 (IF staining)<br>Anti-mouse IgG H&L, Goat-Poly, Colloidal Gold 10 nm (BBI International EM.GMHL10); 5.70×10^12 particles per mL (immunogold)
Validation	Commercial antibodies were validated by the respective suppliers.<br><br>Synechocystis anti-CurT primary antibody (raised against 28-TDVGPITTPNPQKS-41 of Synechocystis CurT) was provided by Dario Leister (LMU Munich), published in Armbruster et al., 2013 (DOI: 10.1105/tpc.113.113118), and previously used in Dann et al., 2021 (DOI: 10.1038/s41477-021-00904-2). Reactivity and specificity was demonstrated previously by immunoblotting of wild-type and curT knockout mutants complemented with AtCURT1A, where the signal was absent in mutant extracts. No cross-reactivity with other Synechocystis proteins was detected previously or in this study.<br><br>anti-AtFTSZ2-1 primary antibody (published in Nakanishi et al., 2009; DOI: 10.1016/j.cub.2008.12.018) wwas provided by Shin-ya Miyagishima. Reactivity against CrFTSZ1/2 was validated by western blot analysis in the course of this study using recombinant CrFTSZ1 and CrFTSZ2 (see Extended Data Fig. 18).

Dual use research of concern

Policy information about [dual use research of concern]

Hazards

Could the accidental, deliberate or reckless misuse of agents or technologies generated in the work, or the application of information presented in the manuscript, pose a threat to:

No	Yes	
☒	☐	Public health
☒	☐	National security
☒	☐	Crops and/or livestock
☒	☐	Ecosystems
☒	☐	Any other significant area

Experiments of concern

Does the work involve any of these experiments of concern:

No	Yes	
☒	☐	Demonstrate how to render a vaccine ineffective
☒	☐	Confer resistance to therapeutically useful antibiotics or antiviral agents
☒	☐	Enhance the virulence of a pathogen or render a nonpathogen virulent
☒	☐	Increase transmissibility of a pathogen
☒	☐	Alter the host range of a pathogen
☒	☐	Enable evasion of diagnostic/detection modalities
☒	☐	Enable the weaponization of a biological agent or toxin
☒	☐	Any other potentially harmful combination of experiments and agents

Plants

Seed stocks	Chlamydomonas reinhardtii strain 137c was used as a wild-type and as a host of generating the curt1a curt1b curt1c triple mutant by CRISPR/Cas9 system. Strain 137c is stocked at the Chlamydomonas Resource Center (http://www.chlamycollection.org) as CC-125.
Novel plant genotypes	The curt1a mutant was generated by CRISPR/Cas9 system using guide RNA CTTTGTCCTACAGAAGCTGG. The curt1b mutant was generated by CRISPR/Cas9 system using guide RNA CCTCCGTGGCTCTGCCCAAG. The curt1c mutant was generated by CRISPR/Cas9 system using guide RNAs TTACTTGCGCGCGGGCAGAG and GCACAGGGGTGTCCGCGCCG. The curt1a curt1b curt1c triple mutant was generated as follows. The curt1a curt1b double mutant was first generated by crossing the curt1a and curt1b mutants and the resultant double mutant was crossed with the curt1c mutant to generate the curt1a curt1b curt1c triple mutant. The triple mutant strain back-crossed twice to the wild-type strain were used in the experiments.
Authentication	The mutants produced by CRISPR/Cas9 system were designed to contain tag sequence at the mutation sites (Fig.3C white rectangles). The mutation can therefore be confirmed by PCR using primers that anneal to sequences upstream or down stream of the mutation site and primers that anneal to tag sequence.

Flow Cytometry

Plots

Confirm that:

- ☒ The axis labels state the marker and fluorochrome used (e.g. CD4-FITC).
- ☒ The axis scales are clearly visible. Include numbers along axes only for bottom left plot of group (a 'group' is an analysis of identical markers).
- ☐ All plots are contour plots with outliers or pseudocolor plots.
- ☐ A numerical value for number of cells or percentage (with statistics) is provided.

Methodology

Sample preparation	Synechocystis and Synechococcus cells were harvested from fresh agar-plate cultures and suspended in liquid BG11 media for analysis. Chlamydomonas reinhardtii cells, in the mid-logarithmic growth phase and maintained in Tris-Acetate-Phosphate (TAP) medium under continuous light conditions, were similarly prepared. The specific details of these procedures are meticulously described in the Materials and Methods section of the manuscript.

Instrument	Analysis was performed using the Muse Cell Analyzer from Millipore.
Software	Software for Data Collection: Muse® software. (ver. 1.8)<br>Data Extraction: Floreada.io. (https://floreada.io/)<br>Calibration Curve for Cell size estimation: Igor Pro 9 (ver. 9.05).<br>Visualization: Python 3.10 with the "python3-mpl-scatter-density" package.
Cell population abundance	Two distinct populations were identified based on red fluorescence levels, which correlate with chlorophyll and/or phycobilin protein content. The high-red fluorescence population represents intact cells, while the low-fluorescence population likely represents cellular debris.<br>The mean cell population abundance, as a percentage of total cell counts, was followings:<br>Synechocystis sp. PCC 6803: approximately 60% for all strains<br>Chlamydomonas reinhardtii: approximately 70% and 80% for WT and curt1abc KO, respectively
Gating strategy	Our flow cytometry analysis employed a gating strategy designed to accurately identify and quantify specific cell populations based on predetermined red fluorescence intensities (30 (Gain 15.3) for Cyanobacteria and 200 (Gain 36.4) for Chlamydomonas), effectively excluding debris.

☐ Tick this box to confirm that a figure exemplifying the gating strategy is provided in the Supplementary Information.
